# Predicting axial muscle-fibre strains in larval zebrafish

**DOI:** 10.1242/jeb.251951

**Published:** 2026-08-12

**Authors:** Johan L. van Leeuwen, Noraly M. M. E. van Meer, Martin J. Lankheet

**Affiliations:** Experimental Zoology Group, Department of Animal Sciences, Wageningen University, De Elst 1, 6708 WD Wageningen, The Netherlands

**Keywords:** Muscle architecture, Strain, Swimming, Larval fish, Mechanical model, Development

## Abstract

The fast axial muscle fibres of teleosts enable rapid starts and high-speed swimming. Their arrangement in densely packed nested helices, combined with shear deformation, has been proposed to reduce variance in muscle-fibre-aligned strains across transverse cross-sections, enabling similar contributions to power generation. In zebrafish (*Danio rerio*), a helical pattern is already present at hatching, but its effect on strain variance remained unclear. Previous studies analysed only the anal region and used distortion-prone vibratome sections. Here, we predicted muscle-fibre-aligned strain distributions as a function of lateral curvature using *in vivo* measurements of muscle-fibre orientation along the anterior 70% of the axial muscles of 4 days post-fertilisation larval zebrafish. Using a bending-beam model, we compared strain distributions for hypothetical longitudinal fibre orientations and the measured helical arrangement, and examined how added-shear deformation and modest median-plane compression alter these distributions. Relative to longitudinal fibres, the helical arrangement reduced muscle-fibre-aligned strains in lateral regions but had little effect on strain variance across transverse sections. Incorporating shear into the helical arrangement, however, consistently reduced strain variance irrespective of body curvature, with the strongest effects anteriorly where helicity is most developed. Allowing median-plane compression further reduced strain variance on the concave side but increased it on the convex side. To assess functional relevance, we predicted maximum mean fibre strains and strain rates along the body from previously recorded swimming kinematics. We conclude that a helical muscle-fibre arrangement with added shear reduces strain variance and supports feasible strain rates during vigorous swimming. These results probably generalise across bony fish larvae.

## Teaser

**Summary:** The helical muscle-fibre architecture in swimming muscle of larval zebrafish is predicted to reduce strain variability in combination with internal shear deformation, which supports swimming at high tail-beat frequencies.

## INTRODUCTION

Larval bony fish belong to the smallest free-swimming vertebrates and usually start to swim shortly after hatching. They face high predation pressure and need to start capturing prey within days, before their yolk runs out. Because of their small size, larval fish operate in the intermediate hydrodynamic regime and must overcome a relatively large viscous drag (McHenry and Lauder, 2005; Li et al., 2016, 2021; Voesenek et al., 2018). To compensate for this high drag, they swim at high tail-beat amplitudes during fast starts, turning manoeuvres and (approximately) cyclic swimming. Additionally, larval fish use high tail-beat frequencies during cyclic swimming. Larval zebrafish, for example, can swim at about 65 body lengths per second (*ℓ*_body_ s^−1^) with tail-beat frequencies of 95–100 Hz (Müller and Van Leeuwen, 2004; Van Leeuwen et al., 2015).

Most fish larvae use their axial muscles to power swimming, which have to meet the challenges of generating high body curvatures at high frequencies. This requires a series of adaptations at multiple architectural levels. First, the neural pattern generator is able to generate the high-frequency activation signals. Second, activation delays along muscle fibres are small as a result of the very short muscle-fibre lengths (114.4±23.3 µm in larval carp *Cyprinus carpio* of 4 days post-hatching and ∼6.7 mm total body length; Alami-Durante et al., 1997). Third, myofibrils, e.g. in herring larvae, have relatively small diameters (about 0.3 µm; Vieira and Johnston, 1992), which favours rapid activation and deactivation by shortening diffusion distances of Ca^2+^ ions. Finally, fish larvae have special, very fast larval muscle-fibre types (Buss and Drapeau, 2000; Mead et al., 2020). These fibre types can generate the required rapid activation and deactivation of the muscles, and the relatively high strain rates, previously estimated to be as high as 30 muscle-fibre lengths per second in fast muscle fibres of zebrafish larvae (Müller and Van Leeuwen, 2004). The muscle fibres express specific larval variants of various molecules, such as Ca^2+^-binding parvalbumin, and myosin heavy- and light chain proteins, which overall support very fast contractions (Scapolo et al., 1988; Martinez et al., 1991; Focant et al., 1992; Veggetti et al., 1993; Mascarello et al., 1995; Schiaffino and Reggiani, 1996; Perry, 1998; Focant et al., 2000, 2003; Hsiao et al., 2003).

**Table:** 

**List of symbols and abbreviations**	
*A*_0_	*A*_0_=*x*_0_d*z*; Fig. 2, Eqn S12
d*ℓ*_f_	Length of a muscle-fibre element in a tissue slice; Eqn S33
d*ℓ*_f0_	As above, but for κ^=0; Eqn S9
d*x*_0_	Distance covered by a muscle-fibre element in a tissue slice in the transverse direction, with κ^=0; Eqn S6
d*y*_0_	As above for dorsoventral distance; Eqn S8
d*z*	Thickness of a transverse tissue slice in the reference configuration with a straight body
d*z*_mp_	Thickness of a transverse tissue slice at the median plane (d*z*_mp_≤d*z*); Eqn 10
*ℓ*_body_	Body length of fish
*ℓ*_m_	Length of the axial muscles
*p*	Exponent used to compute γ distribution; Eqn 4
*p*_opt_	As above, but for the η_opt_ state
*q*	Exponent used to compute γ distribution; Eqn 5; *q*=0.1 in this paper
*R*, R^	Radius of lateral curvature of the median plane; and normalised *R*, calculated by dividing by the maximum half-width of the axial musculature
*S*	Transverse tissue slice
**v**_f_=(*v_x_*,*v_y_*,*v*_z_)	Unit direction vector of a muscle fibre in the fish frame
*X*,*Y*,*Z*	Cartesian frame
*x*,*y*,*z*	Coordinates in the fish-bound reference frame
(*x*_f_,*y*_f_,*z*_f_)	Centre-of-mass location of a muscle fibre
*x*_0_,*y*_0_	The most medial location where a muscle fibre crosses the tissue-slice boundary
*x*_max0_	Maximum width of a unilateral tissue slice in the reference configuration with κ=0
*x*_max0,ax_	Maximum value of *x*_max0_ along the axial muscles
*y*_ep_,*y*_hyp_	The dorsoventral locations where the added shear changes sign in, respectively, the epaxial and hypaxial musculature
*y*_min_,*y*_max_	The most ventral and dorsal points in the transverse tissue slice, respectively
*y*_low_,*y*_up_	The lower and upper limits for interval, respectively, with identical sign of the added shear
α	Azimuth, i.e. angle of the component of a unit direction vector in the frontal plane; Eqn 1
β	Elevation, i.e. angle of a unit direction vector with the frontal plane; Eqn 2
γ	Added shear angle; Eqn 4
γ_max_; γ_max,opt_	Maximum shear angle; and γ_max_ optimised for minimum coefficient of variation in muscle-fibre-aligned strain
γ_med_	γ at the median plane
ϵ_f_	Muscle-fibre-aligned strain
${\varepsilon ¯}_{f}$	Mean value of ε_f_ in a transverse tissue slice
ε_mp_; ε_mp,min_	Longitudinal strain in the median plane; and ε_mp_ at κ^max; Eqn S35
${\varepsilon ¯}_{\mathrm{opt}}$	Mean muscle-fibre-aligned strain in a transverse tissue slice for the minimal |η| case
η	Coefficient of variation in muscle-fibre-aligned strain; η=σ_ε_/${\varepsilon ¯}_{f}$
η_opt_	Value of η that is closest to zero
θ	Angle of a muscle fibre with the longitudinal direction (i.e. *Z*-axis)
κ	Lateral body curvature (κ=1/*R*)
κ^;κ^max	Normalised lateral body curvature (κ^=1/R^); maximum value of κ^ used in the simulations
σ_ε_	Standard deviation in muscle-fibre-aligned strain

Despite the impressive range of molecular and cellular adaptations of axial muscles, only a certain range of strain rates can be accommodated. Super-fast muscles in vertebrates typically exhibit reduced power output (Rome and Lindstedt, 1998; Rome et al., 1999). *In vitro* myotomal linear stretching experiments (Mead et al., 2020) indicate, however, that axial muscles from 5 days post-fertilisation (dpf) zebrafish larvae exhibit superfast twitch kinetics and remarkably high net specific power outputs of 134 and 123 W kg^−1^ during cyclic work loops of 80 and 100 Hz, respectively. During undulatory swimming, however, the body bends laterally, resulting in strain and strain-rate distributions that differ substantially from those imposed in the linear stretching experiments. A more detailed understanding of these distributions is essential to elucidate how fish larvae generate their swimming motions.

Muscle power output depends on strain rate; power peaks generally at about 25% of the maximum contraction speed. Tail-beat frequency and amplitude, and thus body curvature, required for energy-proficient swimming are essentially dictated by physics (Li et al., 2021). Given this constraint, how can the strain and strain-rate amplitudes be tuned for optimal performance of the larva's fast muscle fibres? To answer this question, we need to study the axial muscle architecture, because strain- and strain-rate changes during lateral bending of the fish body depend on the orientation of the muscle fibres (Van Leeuwen et al., 2008). Here, we investigated the effect of the muscle-fibre arrangement on the strain distribution in the fast axial muscles during lateral bending of zebrafish larvae.

The axial muscles in bony fish consist of a longitudinal series of myomeres on the left and right sides of the body. The myomeres on each side of the body are interconnected with connective tissue sheets, called the myosepta (e.g. Greene and Greene, 1914; Alexander, 1969; Gemballa and Vogel, 2002). In adult teleosts, the myomeres are folded into one anterior cone and an epaxial and hypaxial posterior cone. The densely packed muscle fibres in a myomere connect two neighbouring myosepta. Most muscle fibres are not oriented parallel to the longitudinal axis of the fish, but have adopted a nested helical arrangement (e.g. Alexander, 1969; Van Raamsdonk et al., 1974; Mos and Van der Stelt, 1982). Deviations of this pattern may occur in posterior segments that relate to functional specialisations such as the transmission of forces to the caudal fin (Altringham et al., 1993; Ellerby and Altringham, 2001; Davies et al., 1995; Swank et al., 1997; Gemballa and Vogel, 2002). In just-hatched larval zebrafish, myomere shapes are simpler than those in adult fish; the myomeres have shallow anterior cones but lack posterior cones (Van Meer, 2024). Recently, it has been shown that in zebrafish larvae, the nested helical arrangement is probably already present before hatching, and the 2 dpf post-hatching pattern is essentially sustained during larval development (Van Meer et al., 2024, 2026).

**Fig. 1.**
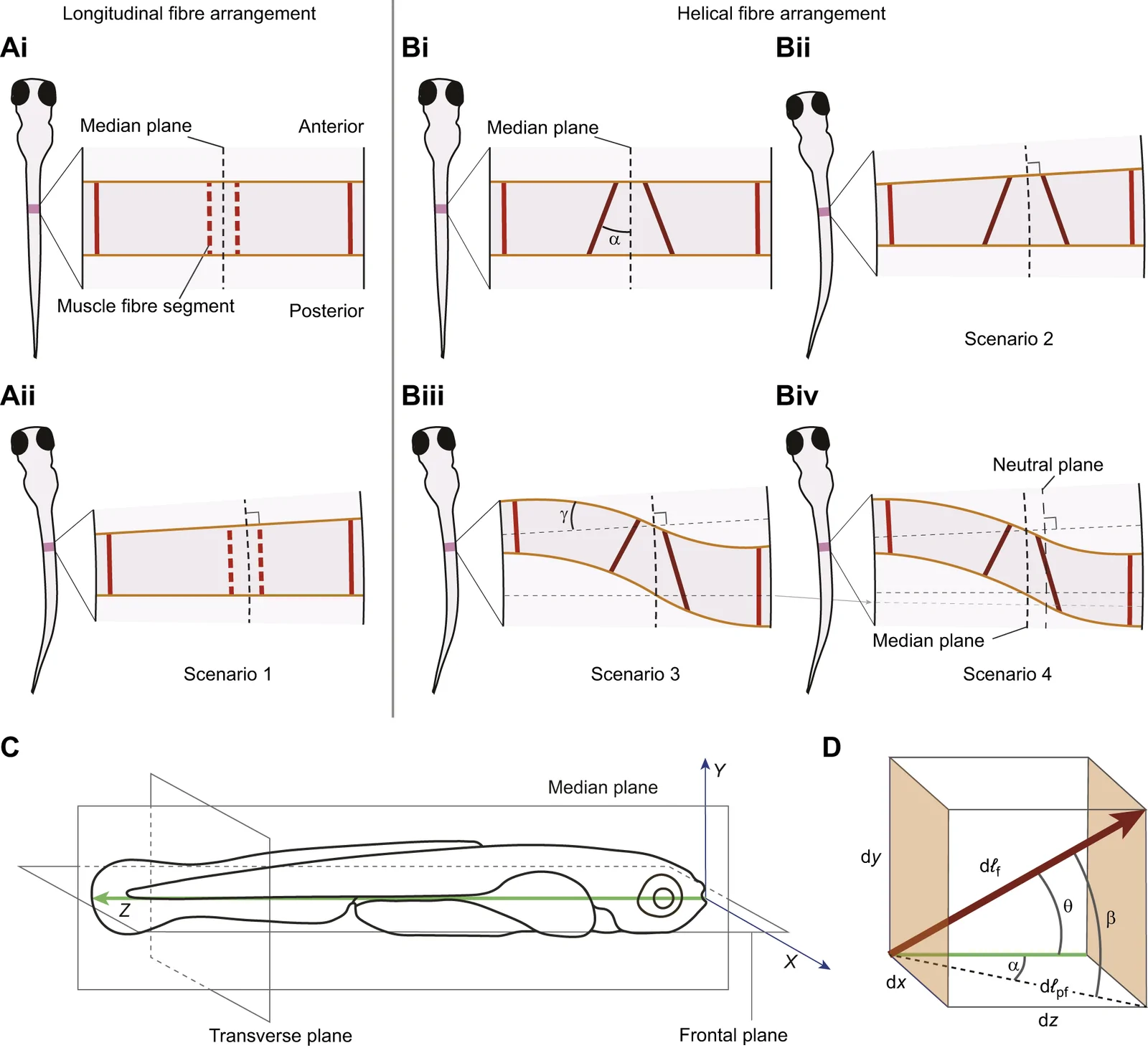
**Effects of added shear on muscle-fibre strain, the Cartesian reference frame and muscle-fibre angles.** (A,B) Schematic dorsoventral views of a muscle segment. The central dashed lines represent the longitudinal axis of the fish, located in the median plane. Orange lines indicate the anterior and posterior boundaries of a muscle-fibre segment; they do not represent myosepta. Dark and light red lines represent medial and lateral muscle-fibre segments. (Ai) Arrangement at rest with a straight median plane and all muscle fibres oriented parallel to the longitudinal axis (zero azimuth). (Aii) Scenario 1: bending towards the left side with all muscle fibres oriented parallel to the longitudinal axis, without added shear deformation. At the concave and convex body sides, large differences in muscle-fibre strain occur between medial and lateral fibre locations. (Bi) Arrangement at rest with a straight median plane and muscle fibres oriented in a helical pattern. Fibre azimuth is indicated by α. (Bii) Scenario 2: bending without added shear deformation. Shortening and lengthening of the medial fibres at the concave and convex sides, respectively, is very small, while substantial mediolateral strain differences occur at each body side. (Biii) Scenario 3: fibre-tension-induced shear deformation during bending, quantified by the shear angle γ. The shear deformation results in an anterior shift of muscle tissue relative to the longitudinal axis on the concave side, and a posterior shift on the convex side. As a consequence, the initially straight orange lines become curved during deformation. At the concave side, fibres near the median plane shorten more than in scenario 2 at the same location, and vice versa at the convex side. (Biv) Scenario 4: similar deformation to that in Biii, but now including longitudinal compression of the median plane (visualised by the transition of the thin grey to the black dashed line) and a shift of the neutral plane (long-dashed line) towards the convex side. Ai,ii and Bi–iii are based on Van Leeuwen et al. (2008). (C) Illustration of the Cartesian reference frame. (D) Definition of muscle-fibre angles: azimuth (α), elevation (β) and angle with the *Z*-axis (θ). The red arrow represents a muscle-fibre segment of length d*ℓ*_f_, and d*ℓ*_pf_ is its projection on the frontal plane.

The functional relevance of the nested helical arrangement has been discussed by various authors (e.g. Van der Stelt, 1968; Alexander, 1969; Gemballa and Vogel, 2002; Van Leeuwen et al., 2008). The prevailing hypothesis suggests that the helical arrangement reduces the segmental heterogeneity – specifically the coefficient of variation – of muscle-fibre-aligned strain along the axial musculature during side-to-side body bending. Several studies have used a theoretical deformation model to estimate muscle-fibre-aligned strains. The simplest approach assumes volume conservation in combination with a relatively simple beam deformation, and a median plane that can bend but does not change in length or height. In such a model, the fibre-aligned strain in muscle fibres oriented parallel to the body axis would be very sensitive to the distance from the median plane during lateral body bending; see Fig. 1A for a planar illustration of this effect. The longitudinally oriented fibres (indicated by the red dashed lines in Fig. 1A) close to the median plane (indicated by the central dashed line) shorten or lengthen very little when the body curves (Fig. 1Aii), compared with the longitudinal lateral fibres (red solid lines). Thus, near the median plane, muscle-fibre-aligned strain would remain minimal as a result of the zero-strain condition of said plane. Strains would increase with distance from the median plane and be highest directly under the skin. The resulting very low strain rates adjacent to the median plane would substantially compromise the overall power output by the axial muscles.

Alternatively, when a muscle fibre near the median plane has a small elevation (angle with the frontal plane; β in Fig. 1D) but a large azimuth (angle between its projection on the frontal plane and the longitudinal axis; α in Fig. 1D), strain limitations may be mitigated if shear deformation in the local (para)frontal plane is considered (Van der Stelt, 1968; Van Leeuwen et al., 2008). Because added shear primarily affects muscle fibres with a substantial azimuth relative to the longitudinal axis, such fibres can undergo large added-shear-related changes in length, whereas longitudinally oriented fibres cannot. In this configuration, actively shortening oblique fibres generate force components along the longitudinal axis that displace local tissue anteriorly or posteriorly relative to the notochord, thereby producing added shear beyond that associated with pure bending. In the planar schematic diagrams of Fig. 1A,B, such oblique fibres (medial dark red lines) therefore change length far more than medial longitudinal fibres (dashed red lines) because they are assumed to induce shear during shortening, quantified by the shear angle γ.

Shear in a (para)frontal plane most strongly reduces fibre-aligned strain at the concave side when the fibre is parallel to that plane; the effect declines with increasing elevation as a result of the added out-of-plane component (Fig. 1D). In larval fish, azimuths near the median plane – at the dorsoventral level of the helix centre – are typically small (Van Meer et al., 2024), probably limiting the predicted strain reduction for helical fibre patterns. Laterally, high elevations, typical of the helical pattern, can offset the strain-increasing effect of median-plane distance. Yet, it remains unresolved whether physiologically feasible shear deformation can substantially reduce fibre-aligned strain variation across the transverse plane.

At present, the extent of median plane strain in larval fish is unknown, largely because it is extremely difficult to measure. During vigorous swimming, axial muscle fibres are expected to impose substantial longitudinal compressive loads on the notochord, making some degree of compression mechanically plausible. The magnitude of this compression will depend on the stiffness of the notochordal sheaths and surrounding tissues. From a functional perspective, limiting notochord compression would require thicker or stiffer sheaths, which would entail increased biological costs associated with their production and maintenance.

In prior strain computations (Van Leeuwen et al., 2008), the median plane was treated as longitudinally incompressible. In this paper, we therefore also explored how modest median-plane compression may affect muscle-fibre-aligned strains. The effect of such compression is illustrated in Fig. 1Biv, where the neutral plane shifts towards the convex side of the body and no longer coincides with the median plane. In this case, longitudinally oriented muscle fibres adjacent to the median plane shorten in concert with the median plane, and muscle-fibre-aligned strain no longer depends solely on the combination of added shear and the fibre azimuth. While this may elevate negative muscle-fibre strains at the concave side, it counteracts muscle-fibre stretching on the convex side.

Most previous studies have not considered variation in muscle-fibre-aligned strain and strain rate along the longitudinal axis of the fish. Van Leeuwen et al. (2008) computed fibre strains only at the anal level using fixed transverse vibratome sections of zebrafish larvae, which unavoidably introduced tissue shrinkage and deformation. In our study, we estimated a densely spaced 3D distribution of muscle-fibre orientations by interpolating a previously collected sparse dataset derived from *in vivo* scans of four 4 dpf zebrafish larvae (Van Meer et al., 2024). At this stage of development, zebrafish larvae do not yet feed but can swim freely.

Here, we used the prescribed-deformation approach to predict muscle-fibre strain and to examine how variation in added shear and longitudinal shortening of the median plane during lateral body bending affects the relative variance in fibre-aligned strain. In the Discussion, we compare this with the alternative, and far more challenging, forward-dynamics approach.

Muscle-fibre-aligned strains were computed using the lateral bending model of Van Leeuwen et al. (2008), which incorporates ‘added-shear’ deformation, and extended here to test additional structural scenarios. Strains were calculated in virtual transverse slices at seven evenly spaced positions along the anterior 70% of the axial musculature, for five different body curvatures. To evaluate the effects of added shear and the nested helical muscle-fibre arrangement, we analysed four structural scenarios. The first three assumed zero median-plane strain: (1) a counterfactual uniform longitudinal fibre orientation (Fig. 1Aii); (2) the reconstructed helical arrangement without added shear (Fig. 1Bii); and (3) the same helical arrangement with added shear (Fig. 1Biii). In scenario 4, we extended scenario 3 by introducing curvature-proportional longitudinal compression of the median plane (Fig. 1Biv). For each transverse-slice–curvature combination in scenarios 3 and 4, we determined the added-shear distribution that minimised strain variation, and from these profiles derived the muscle-fibre-aligned strain distributions and their mean values.

Finally, we predicted maximum strain and strain-rate amplitudes during near-cyclic swimming using body-curvature envelopes obtained from nine previously recorded swimming bouts of 4 dpf zebrafish larvae (Van Leeuwen et al., 2015).

## MATERIALS AND METHODS

Below, we use the shorthand MATLAB to refer to MATLAB 2023b, and the shorthand ‘muscle-fibre strain’ to denote the muscle-fibre-aligned strain component.

### Original data

For this theoretical study, we used previously collected data on the morphology of the axial musculature (Van Meer et al., 2024) and swimming kinematics of 4 dpf larval zebrafish (Van Leeuwen et al., 2015).

### Definition of the fish-bound reference frame

We used a fish-body-attached Cartesian reference frame (Fig. 1C) as applied previously by Van Leeuwen et al. (2008). The positive *Z*-axis is defined by the line between the tips of the snout and the tail, and points posteriorly. The positive *X*-axis points in the right-lateral direction, and the positive *Y*-axis points dorsally. Additional reference frames have been used for various computations (see Supplementary Materials and Methods), which will be introduced where they are first needed.

### Reconstruction of a 3D vector field of muscle-fibre orientation

We used an ensemble dataset of reconstructed muscle-fibre orientations in the axial musculature of four 4 dpf larval zebrafish collected by Van Meer et al. (2024). In that study, the dorsoventrally curved notochord was mathematically straightened, and fibre locations and orientations were defined relative to this virtual straight axis. For the present analysis, however, fibre orientations must be related to the actual body geometry during bending. Because the straightened notochord does not coincide with the instantaneous bending axis of the body – particularly in the anterior region – we transformed all fibre locations and orientations back into a body-fixed Cartesian coordinate system (*X*,*Y*,*Z*) aligned with the undistorted body geometry and lateral bending kinematics. The formal transformation procedure is described in Supplementary Materials and Methods (section S2).

The muscle-fibre data represent an irregularly sampled, sparse dataset, which we first interpolated to obtain a regular grid. Thus, we first defined an equidistant 3D grid with the MATLAB function *meshgrid* that overlapped with the reconstructed raw dataset. We used 300 grid points over the length of the axial muscles of the fish and applied the same resolution in the *X*- and *Y*-directions. For interpolation onto the grid, we used the MATLAB function *griddata*, and the triangulation-based natural neighbour method, without any additional smoothing. To prevent interpolation artefacts in regions with insufficient data support, particularly near the boundaries of the axial muscle volume, we separately interpolated the epaxial and hypaxial muscles. The valid interpolation domain was defined using the MATLAB *alphaShape* and *inShape* functions, ensuring that only anatomically supported regions were retained. Thereafter, these functions served to remove boundary artifacts along the medial and lateral muscle boundaries. We excluded the posterior 25% of the axial musculature from further analysis because of the relatively low number of successfully segmented muscle fibres, which made the interpolation unreliable. For the strain computations, we selected seven transverse sections along the axial musculature, located at 0.1, 0.2, …, 0.6, 0.7 of the axial muscle length (see Fig. 3); section 6 corresponds approximately to the anal position.

Each grid location, (*x*_f_*,y*_f_,*z*_f_), defines the centre of mass of an interpolated ‘virtual’ muscle fibre, and **v**_f_=(*v_x_*,*v_y_*,*v_z_*) is the unit direction vector of the fibre with components in the lateral, dorsal and posterior direction, respectively (Fig. 1D). The azimuth α is defined as the angle of the vector projection in the frontal plane with the longitudinal axis:

(1) $$\alpha =\arctan(v_{x}/v_{z}),\;v_{z}>0.$$

The elevation β is defined as the angle of the fibre with the frontal plane:

(2) $$\beta =\arctan\left(v_{y}/\sqrt{v_{x}^{2}+v_{z}^{2}}\right).$$

Together, α and β define the orientation of a muscle fibre. Finally, the angle θ between the longitudinal direction of the muscle fibre and the *Z*-axis was calculated as:

(3) $$\theta =\arccos\left(v_{z}/\sqrt{v_{x}^{2}+v_{y}^{2}+v_{z}^{2}}\right),\;v_{z}>0.$$

Unlike α and β, θ shows the angular deviation of the muscle-fibre orientation from the longitudinal axis of the fish.

### Calculation of muscle-fibre strain

Muscle-fibre strain depends on: (1) the initial fibre location and orientation in a straight larval fish (the reference shape), (2) an arbitrary lateral curvature of the longitudinal axis of the fish, and (3) certain assumptions about the deformation in the muscle tissue. We mostly present computed strains at the concave body side, but compare these with predicted strains at the convex side. We define the linear, fibre-aligned, strain ε_f_ in a muscle-fibre segment of infinitesimal length d*ℓ*_f_ as (d*ℓ*_f_−d*ℓ*_f0_)/d*ℓ*_f0_, where *ℓ*_f0_ is the resting length of the element. Our computations are based on the approach of Van Leeuwen et al. (2008), which was extended by including the option of median-plane compression. In the Supplementary Materials and Methods (section S3), we provide the detailed mathematical background. Table S1 provides the definitions of the symbols used in the model (Supplementary Materials and Methods, section S1). Below, we outline the most important underlying assumptions and summarise the computational approach.

#### Simplifying assumptions

The following assumptions were made. (i) Muscle tissue is incompressible. Hence, shortening in one direction should be accompanied by lengthening in an orthogonal direction. (ii) The dorsoventral strain in the muscle tissue is zero (called type I deformation by Van Leeuwen et al., 2008), and during lateral bending of the body, this results in lateral thickening at the concave side and thinning at the convex side owing to the incompressibility of the tissue. Van Leeuwen et al. (2008) also considered a type II deformation, characterised by a constant maximum lateral thickness and tissue displacement in the dorsoventral direction. In that study, the computed coefficients of variation in muscle-fibre strain were found to be similar for type I (lateral bulging) and type II (dorsoventral bulging) deformations (see fig. 10 in Van Leeuwen et al., 2008). Fibre-aligned strain in the model is governed primarily by differential endpoint displacement, dominated by added shear near the median plane rather than by the direction of tissue bulging; consequently, mediolateral (type I) and dorsoventral (type II) deformations yield similar qualitative strain-variance outcomes. (iii) We performed computations of muscle-fibre strains under two alternative assumptions: (1) a median plane that bends laterally with radius of curvature *R* without experiencing longitudinal or dorsoventral strain, or (2) a median plane that shortens proportionally with lateral body curvature, while maintaining zero dorsoventral strain. (iv) We computed muscle-fibre strain in seven infinitesimally thin transverse sections along the axial muscles, each assumed to be mechanically independent.

#### Computing muscle-fibre strain

**Fig. 2.**
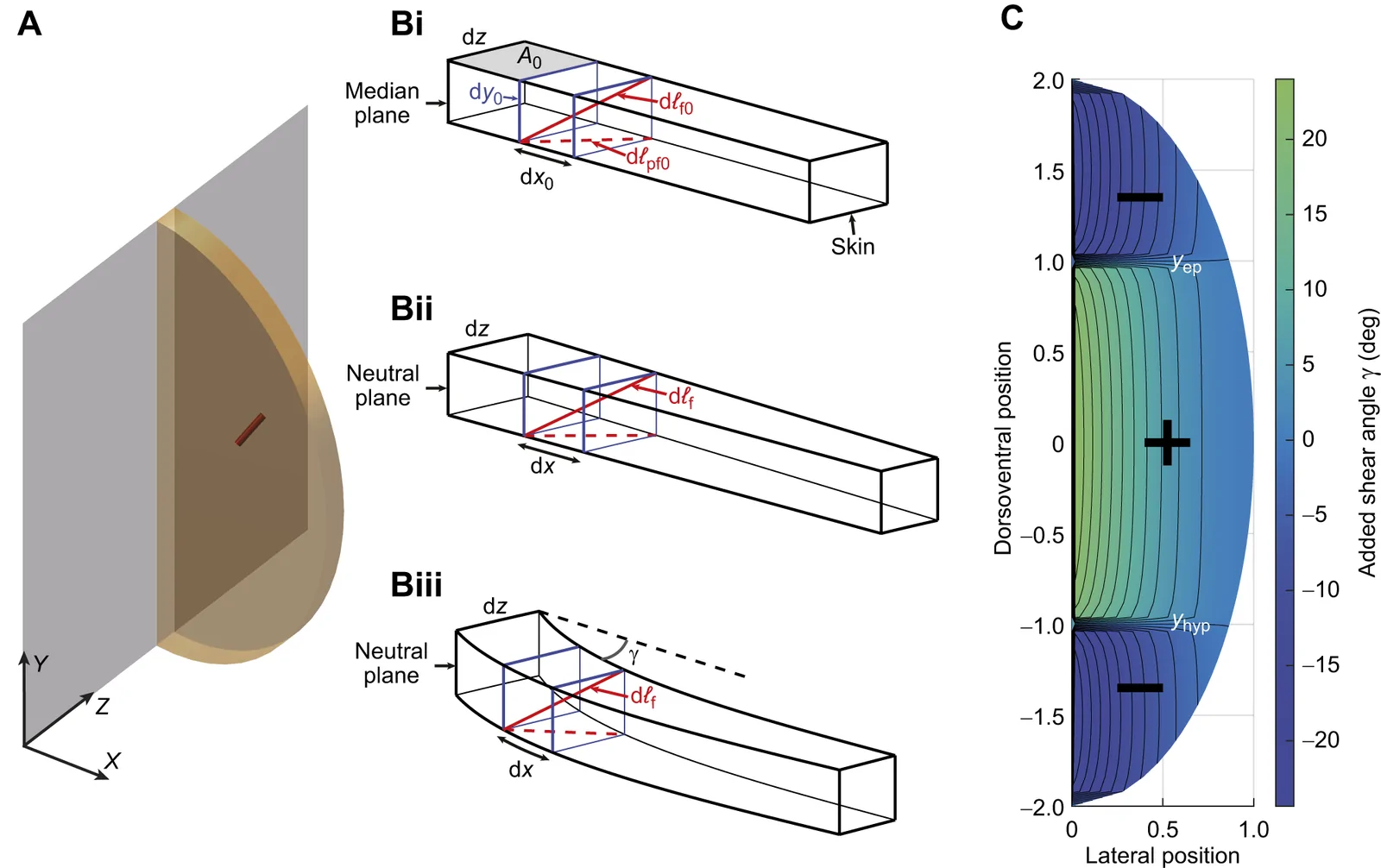
**Diagrams used to explain the computation of muscle-fibre strain in a transverse muscle slice.** (A) Illustration of a portion of the median plane (grey) in the straight reference position, with an attached unilateral transverse muscle slice (brown) of thickness d*z*. The red cylinder in the slice represents an element of a muscle fibre that traverses the slice. (Bi) A mediolateral tissue column (portion of the tissue slice), with a muscle-fibre element (red line). The median plane is straight. Surface *A*_0_ is equal to the distance to the median plane times d*z*. It is used in the strain computations. (Bii) As for Bi, but now with a curved median plane, and without added shear. To preserve volume, the tissue column has elongated in the lateral direction. The width at the median plane is d*z*_mp_, where d*z*_mp_=d*z* for an incompressible median plane and d*z*_mp_≤d*z* for a compressible median plane. (Biii) As for Bii, but now with added shear (γ). (C) Example plot of a γ distribution, with *p*=2.0 and *q*=0.1 (see Eqns 4 and 5). Positions are normalised relative to the local half-width of the body. The bold + and − signs indicate positive and negative added-shear regions. Transitions occur between positive and negative shear at epaxial location *y*_ep_ and hypaxial location *y*_hyp_. Further explanations are provided in the Materials and Methods.

To compute the muscle-fibre strain, we define an infinitesimally thin transverse slice of muscle tissue *S* of thickness d*z* (Fig. 2A). In the reference position, *S* is straight. When the median plane bends, the lateral width of the tissue slice increases and its thickness decreases with increasing distance from the median plane. Together, this preserves the original volume for every infinitesimal element of the slice. Muscle fibres run through *S*; one example is illustrated in Fig. 2A. In the reference position, we computed the length of the muscle-fibre portion in the slice, d*ℓ*_f0_, from its known location, azimuth and elevation (we can ignore the curvature of the muscle fibre because the slice is very thin). The red line in Fig. 2Bi illustrates the position of the fibre element in a transverse column in the tissue slice. Fig. 2Bii illustrates the same column but now for a particular lateral curvature of the median plane. Material points in the column have displaced laterally to preserve volume. We assumed simple bending without added shear here. Using volume preservation, we computed the new length of the muscle-fibre element, d*ℓ*_f_, and strain ε_f_. During swimming, the curvature varies as a function of time and the position along the fish. In this paper, we computed muscle-fibre strains for five prescribed median-plane curvatures and seven locations along the fish (i.e. 35 unique combinations).

As explained in the Introduction, the simple beam deformation restricts muscle-fibre strain adjacent to the median plane, irrespective of the exact orientation of the fibres. However, by adding a particular amount of shear, defined by a prescribed added-shear distribution to the simple beam deformation, muscle fibres can potentially reach sufficiently high strains if they have a large enough azimuth combined with a relatively small elevation. Increasing elevation of the muscle fibre tends to reduce fibre-aligned strain variation because strain in the muscle tissue is assumed to be zero in the dorsoventral direction. This type of deformation is illustrated for the considered tissue column in Fig. 2Biii. Using a meaningful range of added-shear angle γ, we computed *ℓ*_f_ and ε_f_ as a function of body curvature for the considered interpolated muscle-fibre orientations. Following Van Leeuwen et al. (2008), we assumed that γ varies from γ_med_ at the median plane to zero at the maximum width *x*_max0_ of the unilateral tissue slice in the reference configuration according to:

(4) $$\gamma (x,y)=\gamma_{\mathrm{med}}{\left(\frac{x_{\max0}-x_{0}}{x_{\max0}}\right)}^{p},$$

where γ_med_ is defined as:

$$\gamma_{\mathrm{med}}(y)=\pm \gamma_{\max}{\left(\cos\left(-\frac{\pi }{2}+\pi \frac{y-y_{\mathrm{low}}}{y_{\mathrm{up}}-y_{\mathrm{low}}}\right)\right)}^{q},$$

(5) $$y\in [y_{\mathrm{low}}\;y_{\mathrm{up}}]$$

and γ_max_ is the maximum value of γ. This formulation provides a geometrically consistent but flexible description of spatially varying shear across the transverse section. In particular, it allows the shear angle to vary non-linearly with mediolateral and dorsoventral position, so that shear decreases from the median plane towards the skin. The *p*-value determines how rapidly the shear angle decreases from the median plane to the skin. This function enables an exploration of the non-linear variation of γ with distance from the median plane and its effect on ε_f_. We used a fixed value of 0.1 for *q* in our computations. This choice ensures γ_med_ remains nearly constant except near the lower and upper bounds of each interval [*y*_low_ *y*_up_]. The value of γ_max_ is a positive real number and constant for each computation of a strain field for the considered half-body slice.

We distinguish three intervals between *y*_min_ (the ventralmost point) and *y*_max_ (the dorsalmost point) that are based on morphological features: [*y*_min_ *y*_hyp_], [*y*_hyp_ *y*_ep_] and [*y*_ep_ *y*_max_], where *y*_hyp_ and *y*_ep_ are the dorsoventral (hypaxial and epaxial) locations where the medial fibres change direction from positive to negative azimuth or vice versa. These locations deviate slightly from the position of the helix centres, which were taken as *y*_hyp_ and *y*_ep_ by Van Leeuwen et al. (2008). The ± sign in Eqn 5 stands for a minus or plus sign according to the convention of Fig. 2C, which shows an example distribution for *p*=2.0. The sign of expression (Eqn 5) is constant within each region but changes at the transitions between neighbouring regions, reflecting the reversal of fibre azimuth across the dorsoventral axis associated with the helical muscle organisation. Thus, the sign change represents a reorientation of the local shear direction consistent with the underlying fibre geometry, rather than a discontinuity or a change in deformation mode. Any discontinuities in the considered tissue slab at the transitions are avoided because at these locations γ=0 (Eqns 4 and 5). The prescribed shear deformation does not affect the location of the neutral plane.

The absolute shear value is highest near the median plane and lowest near the skin. Between *y*_hyp_ and *y*_ep_, γ is positive at the concave side, which means that the muscle mass shifts anteriorly relative to the median plane. This is based on the predominantly posterolateral direction of the medial muscle fibres – a property of the helix organisation of the epaxial and hypaxial muscles. Dorsal to *y*_ep_ and ventral to *y*_hyp_, γ is negative, which relates to the prevailing posteromedial orientation of the medial muscle fibres.

Because muscle-fibre-aligned strain is the primary output quantity of interest, we focused on reporting computed strains directly rather than intermediate displacement quantities. For the ensemble of muscle-fibre orientations in each of the seven axial muscle slices, we computed the coefficient of variation in fibre strain as:

(6) $$\eta =\sigma_{\varepsilon }/{\varepsilon ¯}_{f},$$

where σ_ε_ is the standard deviation of the strain and ${\varepsilon ¯}_{f}$ is the mean muscle-fibre strain in a tissue slice.

#### Model simulations

We computed strains for the seven selected virtual slices along the axial muscles. We varied the median-plane curvature across and slightly beyond the physiologically relevant range. Curvature is defined as:

(7) κ=1/R,

where *R* is the radius of curvature of the median plane. In the simulations, we normalised κ by multiplication with the maximum unilateral width *x*_max0,ax_ of the axial muscles in the straight reference position:

(8) κ^=xmax0,axκ.

Alternatively, we could have normalised the curvatures by multiplication with the local unilateral width. That approach would, however, complicate comparisons between sections. We performed computations for five evenly spaced values of κ^: 0.04, 0.065, 0.09, 0.115 and 0.14. The value 0.09 slightly exceeds the maximum κ^ observed along the body during vigorous near-cyclic swimming, while 0.14 lies just beyond the peak values seen in extreme turning or fast-start manoeuvres.

We explored four simulation scenarios to put the effects of the reconstructed nested helical fibre orientations and added shear on muscle-fibre strain into perspective. In scenarios 1–3, we assumed an incompressible median plane. In the counterfactual scenario 1, all muscle fibres were assumed to be aligned parallel to the longitudinal *Z*-axis. For longitudinally oriented muscle fibres, added shear produces a uniform translation of the entire fibre segment without differential deformation and therefore does not affect fibre strain. For scenario 2, we used the reconstructed muscle-fibre directions while assuming simple beam deformation without additional shear. In scenario 3, we explored the effect of the form of the added shear distribution, γ(*x*,*y*), on mean muscle-fibre strain and on the coefficient of variation η by varying γ_max_ and exponent *p* (see Eqns 4 and 5). To estimate the maximum effect of added shear on strain-variance reduction, we optimised γ_max_ and *p* (γ_max,opt_ and *p*_opt_) to minimise |η|, at each longitudinal location and normalised curvature. The resulting value is denoted η_opt_. We compared η_opt_ with the corresponding values from scenarios 1 and 2 to quantify the extent to which optimised added shear can reduce relative strain variance. Finally, for scenario 4, we used the settings from scenario 3, but allowed for a slight median-plane shortening proportional to the lateral body curvature. This scenario was included as a robustness test and is not intended to represent a reported swimming condition.

In scenario 4, the longitudinal median-plane strain was computed as:

(9) εmp=κ^εmp,minκ^max,

where κ^max=0.14 is the maximum κ^-value used in the simulations and ε_mp,min_=−0.01 is the corresponding, prescribed, minimum longitudinal strain in the median plane. While the visco-elastic properties of the notochord in adult hagfish have been characterised (Long et al., 2002), we are not aware of studies reporting longitudinal strain in the median plane of the notochord during free swimming, either in larval bony fish or in adult animals that retain a notochord. We therefore restrict the present analysis to small-amplitude median-plane strain. Eqn 10 was subsequently used to compute the tissue-slice thickness at the median plane:

(10) $$dz_{\mathrm{mp}}=dz(1+\varepsilon_{\mathrm{mp}}).$$

### Deriving lateral body curvature and muscle-fibre strain predictions in free-swimming fish larvae

We used the normalised curvature envelope of near-cyclic swimming events and an estimated extreme body curvature used in turning manoeuvres (κ^≈0.13) to approximate the extent of the physiological range. We predicted the corresponding mean muscle-fibre strain amplitudes used in such motions, assuming optimised added shear to achieve minimal relative strain variance.

We used the kinematic data of the central body axis for nine near-cyclic swimming events of 4 dpf larval fish collected by Van Leeuwen et al. (2015, and associated data) to compute lateral body curvature. The reported mean body length was 4.37 mm. Fig. S3 shows body-axis plots of the nine swimming bouts. We slightly smoothed the body axes with a quintic spline function (MATLAB function *spaps*; tolerance setting: 10^−6^), because curvature computations were very sensitive to noise in axis coordinates. Using the central-axis shape, we derived the curvature (κ) along the body. By multiplication with the maximum half-width of the axial musculature (70 µm), we obtained normalised curvature (κ^) as a function of time and location along the body. For 101 regularly spaced locations along the axis, we collected κ^ for all instances of the nine swimming events. Curvatures at the snout and tail tip were set to zero. To remove outliers, we excluded the top 1% of curvature values at each location and used the remaining maxima to define a raw curvature envelope, which was then smoothed using a cubic spline (MATLAB *spaps* function; tolerance setting: 3×10^−5^). The portion of the smoothed curvature envelope that overlapped with the seven muscle slices served as input for estimating the maximum strain generated in the axial musculature during near-cyclic swimming. Fig. S4 illustrates both the ensemble of the computed curvatures and the resulting curvature envelope.

### Use of AI tools

Language editing and clarity improvements were assisted by OpenAI's ChatGPT and Microsoft's Copilot, based on author-provided input and guidance. The MATLAB code was written by the first author and subsequently tested and validated. Final code optimization and testing were supported by Copilot based on author-provided input and instructions. The authors critically reviewed, verified and revised all manuscript and code content as necessary and take full responsibility for the publication's final content.

## RESULTS

### Computed muscle-fibre orientations

**Fig. 3.**
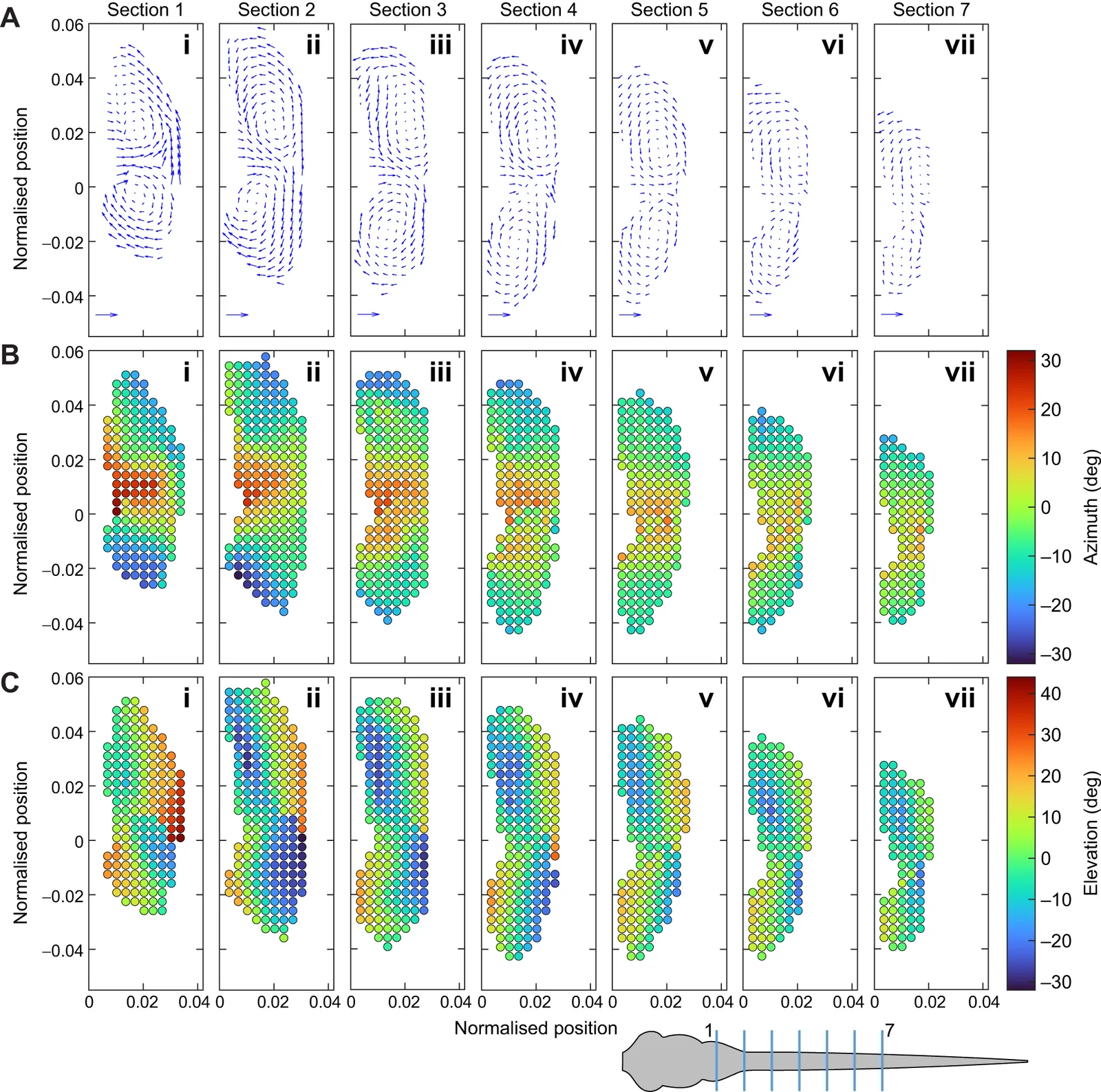
**Muscle-fibre orientations used for strain computation.** Interpolated muscle-fibre orientations are shown at seven regularly spaced transverse sections along the axial muscles (locations 0.1, 0.2, …, 0.7 in normalised coordinates) of a straight 4 days post-fertilisation (dpf) larval zebrafish. The bottom-right pictogram shows the location of the seven sections along the fish. (Ai–vii) Quiver plots of the projections of the muscle-fibre unit vectors on the transverse plane. In the lower-left corner, an in-plane unit vector is shown for reference. (Bi–vii) Computed azimuth (α) at locations corresponding to the vectors shown in Ai–vii. (Ci–vii) As for B, but for the computed elevations (β). Positions are normalised relative to axial muscle length.

Fig. 3Ai–vii shows projections of the reconstructed unit direction vectors of the muscle fibres in seven transverse planes at equidistant positions along the axial musculature, normalised by muscle length (0.1, 0.2, …, 0.7). An approximately helical muscle-fibre arrangement is present in the epaxial and hypaxial muscles. The epaxial and hypaxial patterns are, roughly, each other's mirror image. Overall, the vector projections shorten in the posterior direction (i.e. from section 1 to 7), indicating a decrease in fibre angle with the longitudinal axis. Fig. 3Bi–vii,Ci–vii shows the corresponding azimuth (α) and elevation (β) values, respectively. Anteriorly, the largest (absolute) angles are present, and angles are smallest at the helix centres, where the fibres run nearly longitudinally.

### Strain predictions for longitudinally oriented muscle fibres

**Fig. 4.**
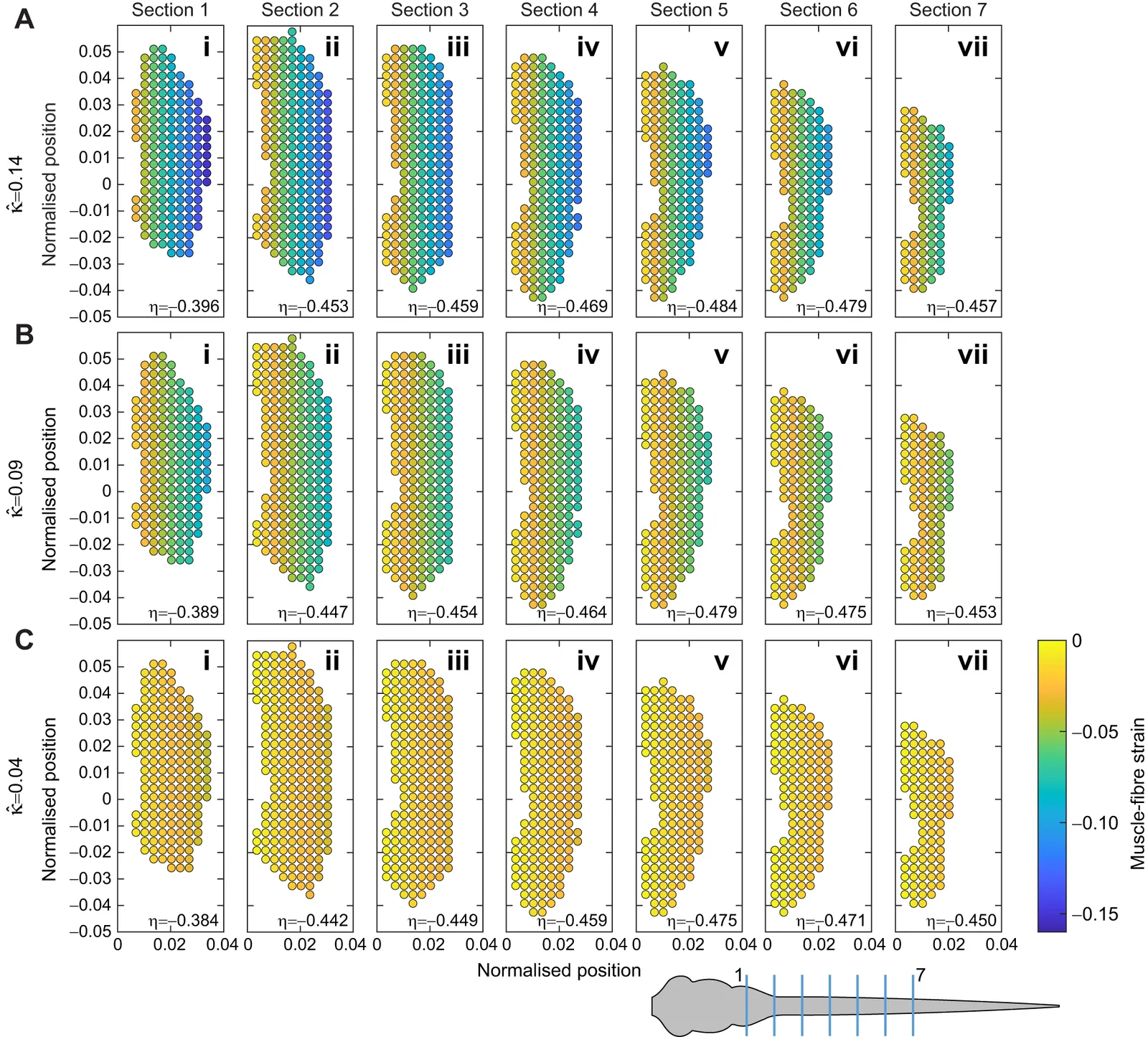
**Computed muscle-fibre strain on the concave side of the body for seven transverse sections along the axial musculature (rows) and three different normalised curvatures (**κ^**, columns), based on scenario 1, in which all muscle fibres are oriented parallel to the longitudinal axis.** The bottom-right pictogram shows the location of the seven sections along the fish. (Ai–vii) Strain for a strong body curvature (κ^=0.14). A negative gradient is seen from left to right; strain decreases with distance from the median plane. The larger the width of the musculature, the more negative the most extreme strain becomes. (Bi–vii) Similar to Ai–vii, but for κ^=0.09. (Ci–vii) Similar to Ai–vii, but for a small curvature (κ^=0.04). The coefficient of variation η in the muscle-fibre strain is shown at the bottom of each panel. Values of |η| are relatively large and increase slightly with curvature.

We first considered two model scenarios without added shear to examine the influence of a helical muscle-fibre arrangement on strain distribution. In the first, counterfactual scenario, all muscle fibres were assumed to be oriented parallel to the body's longitudinal axis (θ=0). Fig. 4 shows the resulting muscle-fibre strain distributions at the concave side of the body, across seven transverse slices along the axial musculature, for three values of normalised curvature κ^. Throughout this study, negative strain values indicate muscle-fibre shortening.

The lowest value (κ^=0.04) represents a slight curvature, typical of amplitudes during slow swimming. The second value (κ^=0.09) is below the amplitudes produced during (sub)maximal turning manoeuvres and (sub)maximal fast starts, and is just above the maximum for fast, near-cyclic swimming (see Materials and Methods subsection ‘Deriving lateral body curvature and muscle-fibre strain predictions in free-swimming fish larvae’). The highest value (κ^=0.14) exceeds the normal physiological range.

Three trends are visible. First, as expected, increasing κ^ leads to more negative strain values, indicating that the muscle fibres shorten to a greater extent. Second, strain decreases from medial to lateral, which is typical for a simple bending beam model. So, medial muscle fibres would shorten less than lateral muscle fibres. For a given κ^, strain decreases at the same rate from medial to lateral for all slices. Fibres adjacent to the median plane can hardly shorten because the median plane maintains its prescribed zero-strain state while it bends. This situation would not change if we were to implement added shear in the beam deformation because the azimuth of longitudinally oriented muscle fibres is zero. This would restrict the potential power and work output of muscle fibres adjacent to the median plane. The resulting absolute values of the coefficient of variation |η| are relatively high (η-range is [−0.48 −0.39]) in the considered parameter space. For all slices, |η| increases slightly with rising κ^. Third, for a given value of κ^, the strain range decreases from anterior to posterior as a result of the diminishing width of the axial musculature, which restricts the lateral position of the muscle fibres. Negative strain peaks at the lateral side in section 1, which has the largest muscle width of all sections. We conclude that a longitudinal muscle-fibre alignment is ineffective in reducing the variance in fibre-aligned strain.

### Strain predictions for reconstructed muscle-fibre orientations without added shear

**Fig. 5.**
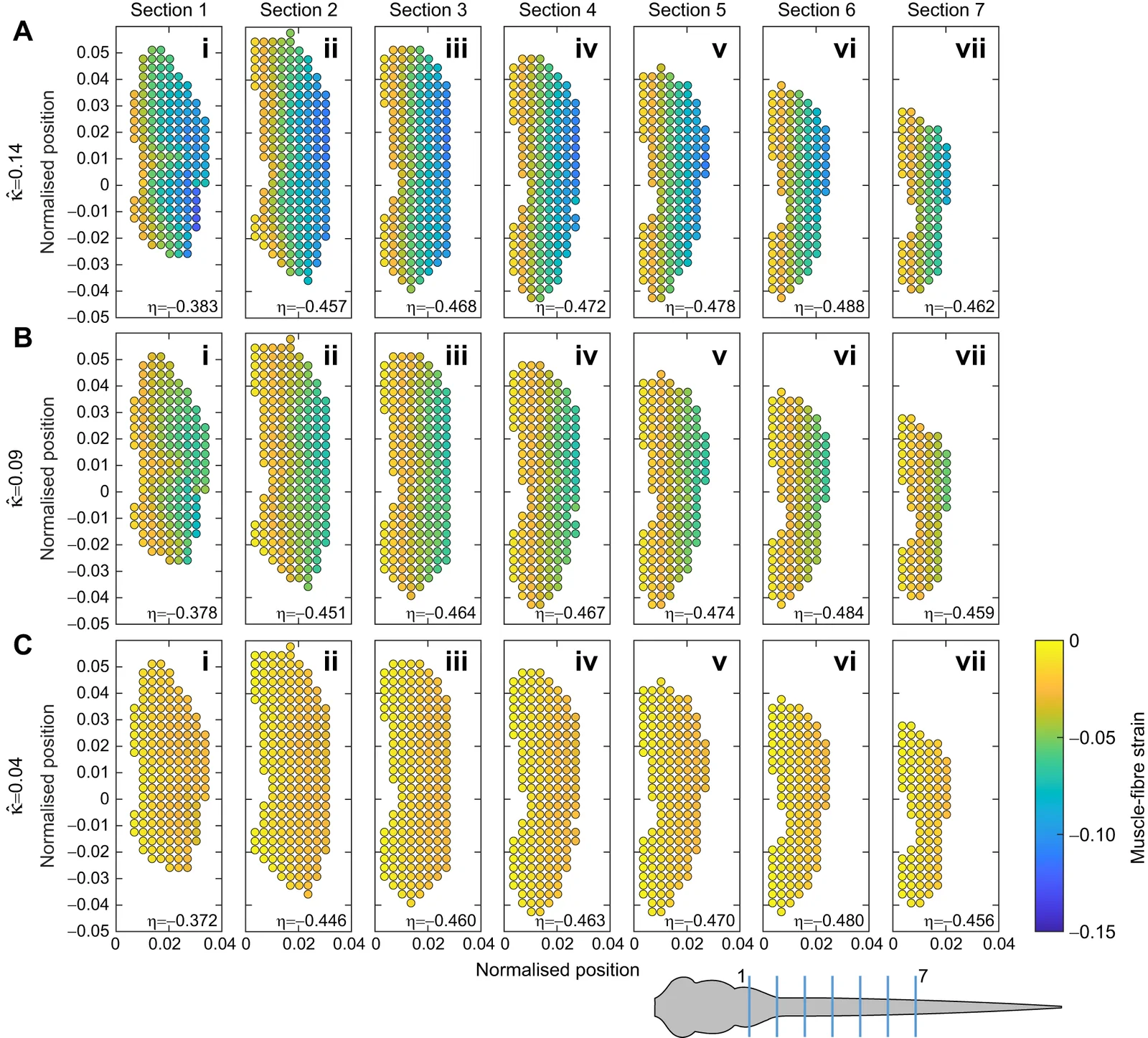
**Computed muscle-fibre strain on the concave side of the body for seven transverse sections along the axial musculature (rows) and three different normalised curvatures (**κ^**, columns), based on scenario 2, in which no added shear is included in the deformation (γ=0).** The bottom-right pictogram shows the location of the seven sections along the fish. (Ai–vii) Strain for a strong body curvature (κ^=0.14). (Bi–vii) Similar to Ai–vii, but for κ^=0.09. (Ci–vii) Similar to Ai–vii, but for a small curvature (κ^=0.04). The coefficient of variation η in the muscle-fibre strain is shown at the bottom of each panel. Values of |η| are relatively large and increase slightly with curvature. Strain tends to increase from medial to lateral, but the pattern is less regular than in scenario 1 with longitudinally oriented muscle fibres (see Fig. 4).

The second scenario takes reconstructed muscle-fibre directions into account but assumes zero added shear. Fig. 5 shows the resulting muscle-fibre strain at the concave body side for the same κ^ values as in Fig. 4. Again, strain increases with κ^ and decreases in the posterior direction for a given κ^ value. Overall, absolute strain |ε_f_| is lower at identical locations and κ^-values than for scenario 1 and also tends to decrease in the medial direction, resulting from local values in azimuth (α) and elevation (β).

For instance, large fibre elevations (i.e. with a large *v_y_* component) at the lateral side (e.g. the lateral epaxial portion of section 1 in Fig. 3), typical of a helical arrangement, reduce muscle-fibre strain because dorsoventral tissue strain is zero (consequence of type 1 deformation). This helps prevent excessive muscle-fibre strain at the lateral side. For the same reason, at the medial side, large elevations – also typical of a helical pattern – further decrease, rather than increase, fibre strain. Large azimuths (and thus large *v_x_* components) at the medial side tend to further reduce the already low strain instead of increasing it, because a (slight) lateral expansion occurs under the set boundary conditions of the body deformation (Fig. 2Bii). For scenario 2, the coefficients of variation resemble those of scenario 1, with a range of [−0.49−0.37] in the considered parameter space. For all slices along the body, |η| increases slightly with increasing κ^.

We conclude that the helical nature of the reconstructed muscle-fibre arrangement helps reduce muscle-fibre strain at the lateral side, but in the absence of added shear, fibre strain at small lateral distances is even more reduced by this spatial arrangement. Without added shear, a helical pattern does not substantially reduce the variance in muscle-fibre strain.

### Strain predictions for reconstructed muscle-fibre orientations with added shear

In scenario 3, we included an added-shear deformation in the muscle-fibre strain computations. To evaluate how the shear distribution influences the strain variability, we analysed the effect of two parameters (see Eqns 4 and 5): γ_max_ (the maximum shear angle) and *p* (which determines how rapidly shear decreases across the mediolateral axis). Specifically, we assessed how variation in these parameters affected the coefficient of variation η in muscle-fibre strain.

**Fig. 6.**
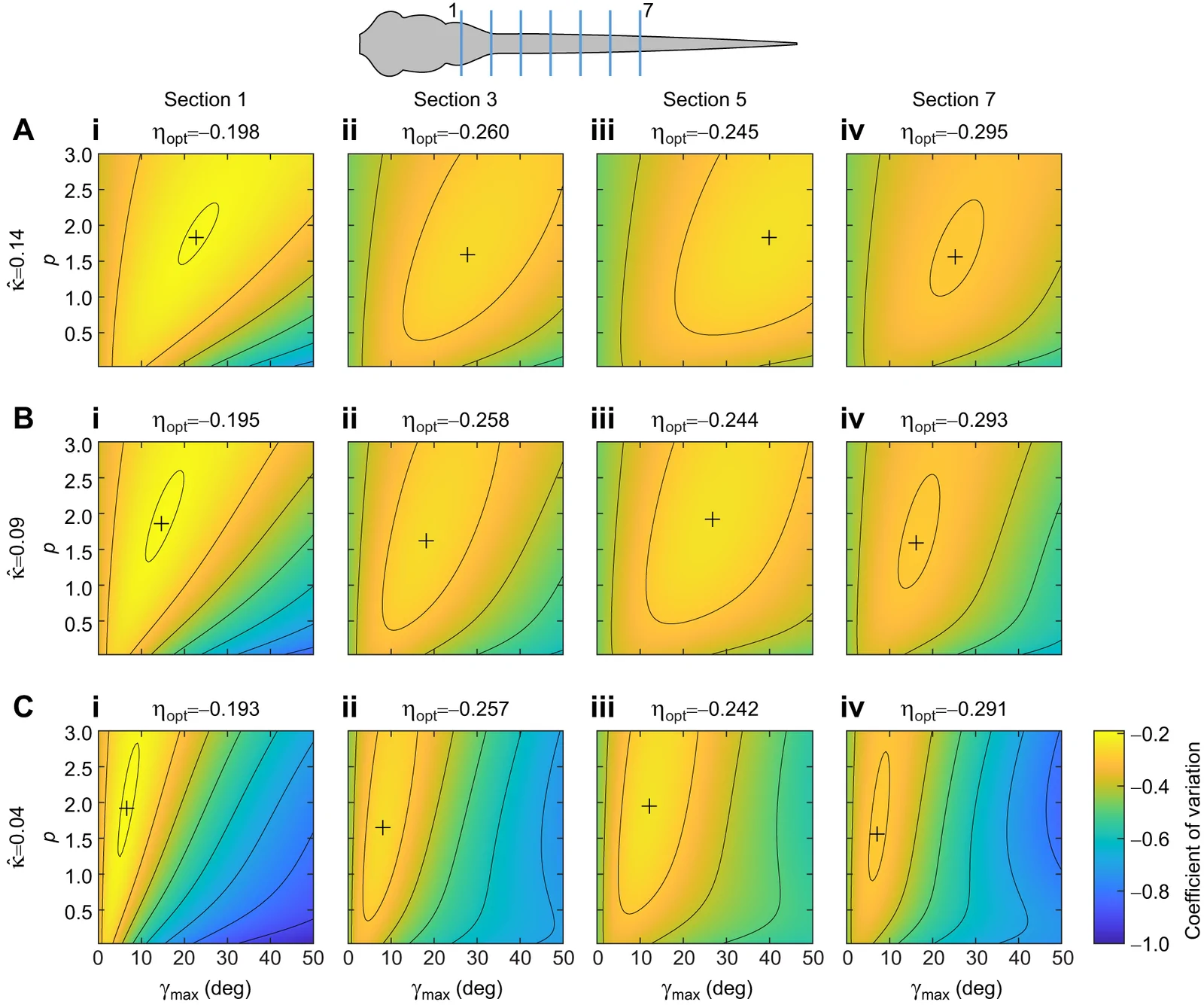
**Contour plots of the coefficient of variation η of computed muscle-fibre strain as a function of γ_max_ and exponent *p* (scenario 3).** Results are shown for the concave side of the body using the reconstructed muscle-fibre arrangement. Each panel shows a unique combination of anteroposterior location (indicated by section number) and normalised curvature (κ^). Sections 1, 3, 5 and 7 are located respectively at 0.1, 0.3, 0.5 and 0.7 of the axial muscle length. The pictogram at the top shows the location of the selected sections along the fish. For each plot, η_opt_ is given; the closer η_opt_ is to zero, the lower is the variation in muscle-fibre strain. The ‘+’ symbol indicates the location of η_opt_ in the (γ_max_,*p*) parameter space.

Fig. 6 presents contour plots of η in the (γ_max_,*p*) parameter space, for transverse slices 1, 3, 5 and 7 along the axial musculature, each evaluated at three levels of normalised curvature κ^. These plots illustrate how relative strain variation depends on the shape of the added-shear distribution. In each panel, the minimum of the contour plot represents the optimal parameter combination that yields the least relative strain variation, denoted as η_opt_ (shown above each panel). The location of this optimum is marked by a ‘+’ symbol, and the corresponding parameter values are referred to as γ_max,opt_ and *p*_opt_. As κ^ increases, γ_max,opt_ also increases, while *p*_opt_ shows relatively little variation. Among the slices, slice 1 exhibits the lowest relative strain variation, and slice 7 the highest. Overall, the observed range of η_opt_ ([−0.30 −0.19]) is much closer to zero than in scenario 2 without added shear (Fig. 5), indicating that a suitable added-shear distribution combined with a helical muscle-fibre arrangement substantially reduces strain variation.

**Fig. 7.**
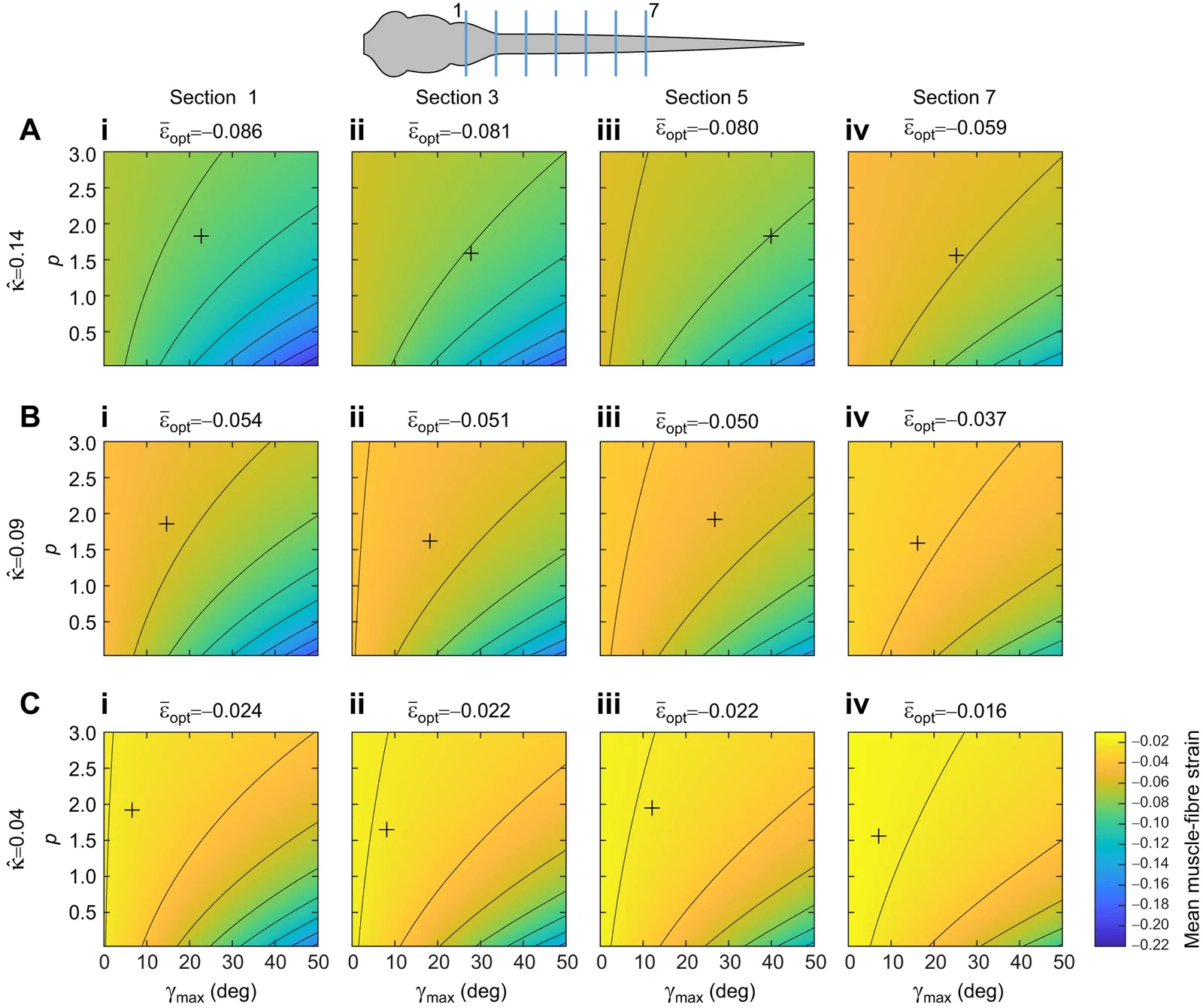
**Contour plots of the mean longitudinal muscle-fibre strain** ${\varepsilon ¯}_{f}$ **as a function of γ_max_ and exponent *p* (scenario 3).** Each panel represents a unique combination of location along the axial musculature (indicated by section number) and normalised curvature (given by κ^). The computations were made for the concave body side. Sections 1, 3, 5 and 7 are located respectively at 0.1, 0.3, 0.5 and 0.7 of the axial muscle length. The pictogram at the top shows the location of the selected sections along the fish. For each plot, ${\varepsilon ¯}_{\mathrm{opt}}$ is given, which is the average strain corresponding to the η_opt_ condition. Fibre strain becomes more negative with increasing curvature. For a given value of κ^, strain magnitude tends to decrease in the posterior direction (from sections 1 to 7). The ‘+’ symbol indicates the location of ${\varepsilon ¯}_{\mathrm{opt}}$ in the (γ_max_,*p*) parameter space, corresponding to η_opt_ (see Fig. 6).

Fig. 7 presents the corresponding contour plots of the mean muscle-fibre strain, ${\varepsilon ¯}_{f}$, for the same transverse slices and normalised curvature (κ^) values used in Fig. 6. In each panel, the ‘+’ symbol marks the location of η_opt_, and the corresponding strain value ${\varepsilon ¯}_{\mathrm{opt}}$ is given on the top. As κ^ increases, the mean strain becomes more negative. Moreover, for a given κ^, the mean strain becomes less negative in more posterior slices, reflecting a decrease in azimuthal fibre orientation and body width.

**Fig. 8.**
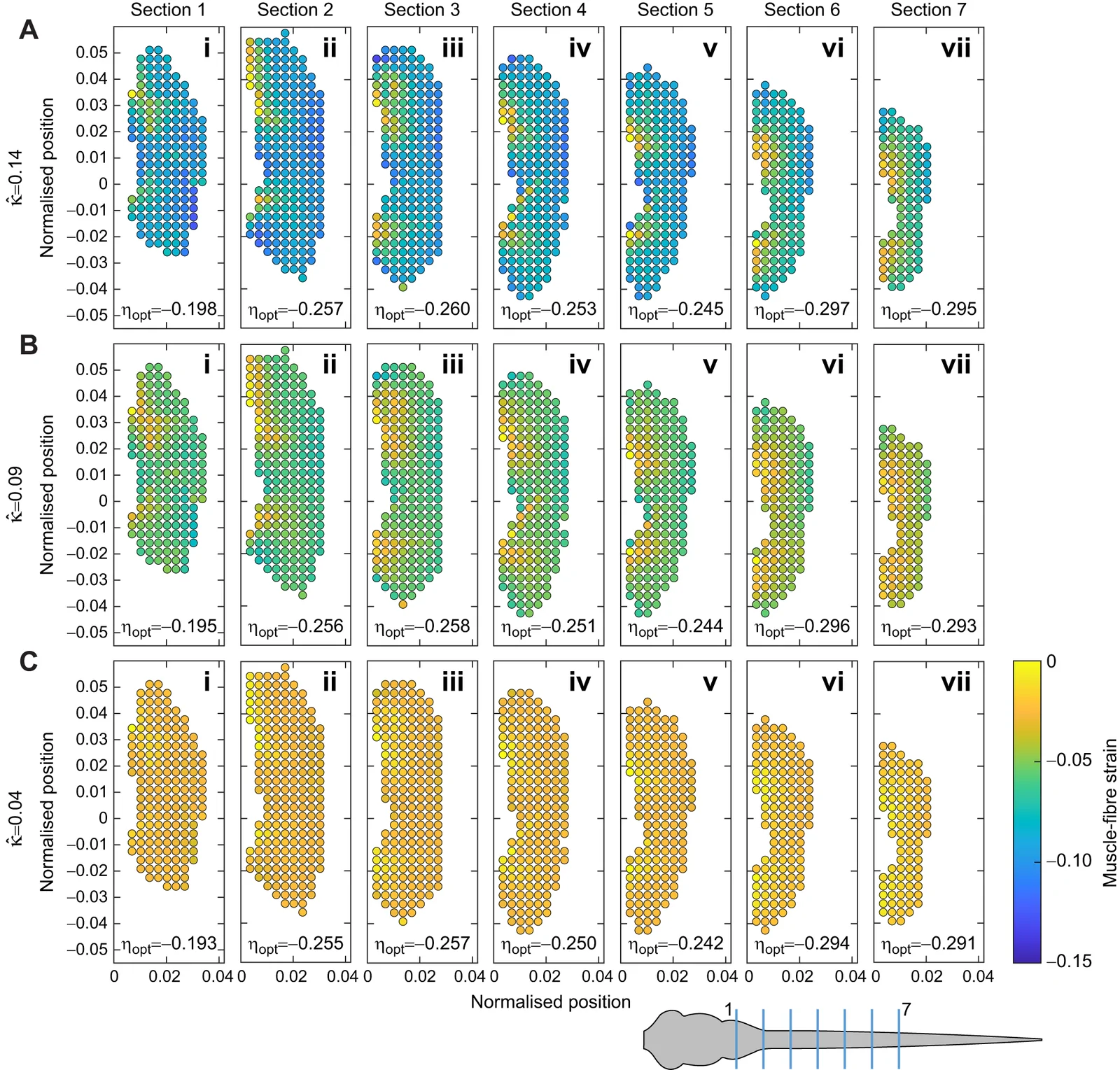
**Computed muscle-fibre strain for the reconstructed muscle-fibre pattern and added shear for the concave body side (scenario 3).** Combinations are shown for seven transverse sections along the axial muscles (rows) and three different normalised curvatures κ^ (columns). The bottom-right pictogram shows the location of the seven sections along the fish. In the computations, the added shear was optimised for minimal strain variation (γ_max_=γ_max,opt_). (Ai–vii) Strain for a large body curvature (κ^=0.14). (Bi–vii) Similar to Ai–vii, but for κ^=0.09. (Ci–vii) Similar to Ai–vii, but for a small curvature (κ^=0.04). The optimised coefficient of variation η_opt_ for muscle-fibre strain is shown at the bottom of each panel. Values of |η_opt_| increase slightly with curvature and are much smaller than those with zero added shear (Fig. 5) or (counterfactual) longitudinally oriented muscle fibres (Fig. 4).

Fig. 8 shows distributions of muscle-fibre strain for the seven tissue slices at the concave side of the body, at three levels of normalised curvature κ^ and minimal muscle-fibre strain variation (η=η_opt_). Again, strain increases with κ^ and decreases in the posterior direction. The clear mediolateral strain gradient of scenarios 1 and 2 is absent for the optimised added-shear case. Instead, the distributions tend to be less variable, with a few hotspots showing outlier strain values. First, some hotspots show low negative strain near the median plane (e.g. dorsomedial position in section 6), where azimuth values are low, causing the added shear to be ineffective. Second, other hotspots show high negative strain at lateral locations (e.g. ventrolateral position in section 1) where low fibre angles with the longitudinal direction reduce an elevation-induced compensation for the positive effect of lateral distance on strain amplitude.

**Fig. 9.**
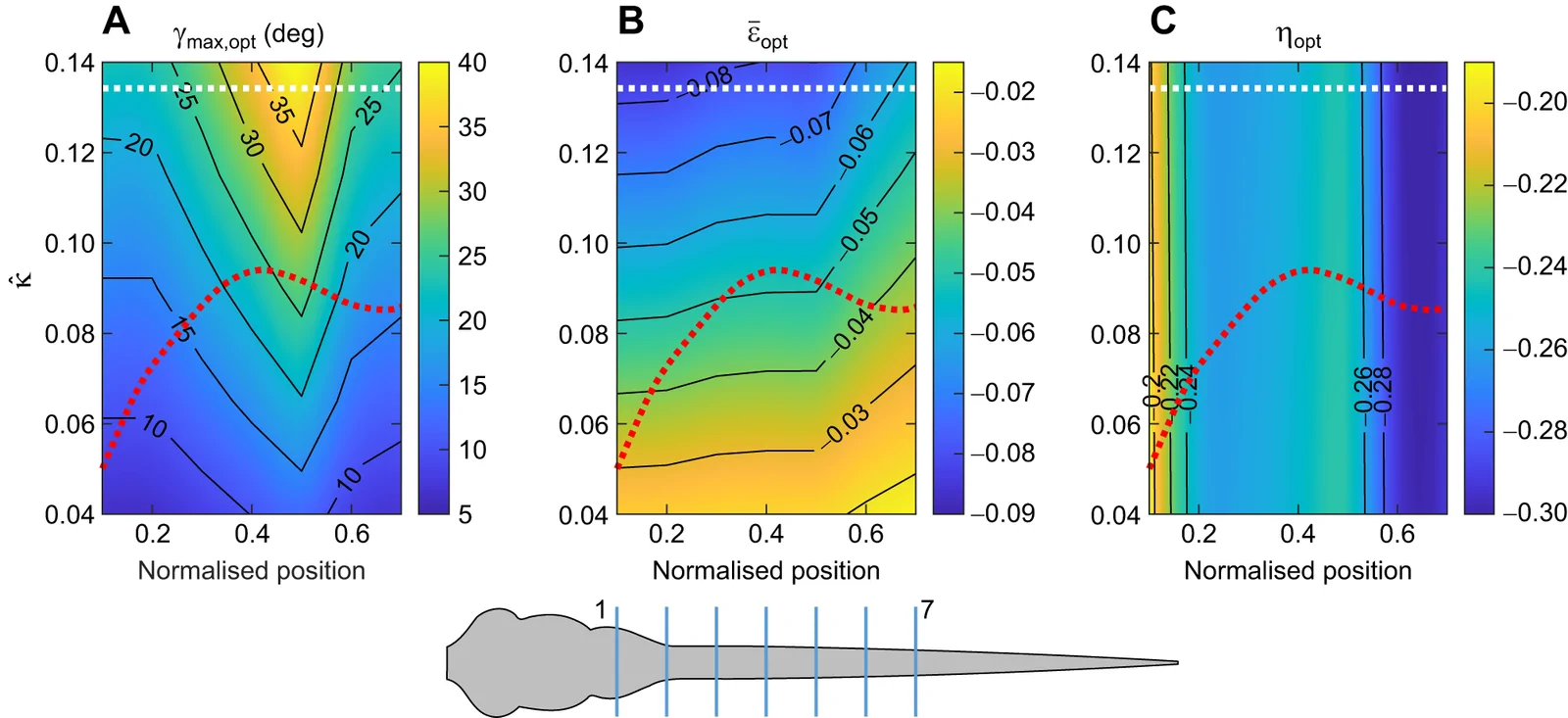
**Overview of the effect of normalised curvature (**κ^**) and location along axial muscles on the optimised maximum added shear (γ_max,opt_), mean muscle-fibre strain (**${\varepsilon ¯}_{\mathrm{opt}}$**) and coefficient of variation (η_opt_) for minimal relative strain variation (scenario 3).** Results are shown for the concave body side. Anteroposterior location is normalised relative to axial muscle length. The pictogram at the bottom shows the location of the seven sections along the fish that were used to generate the contour plots. Contour plots are shown of γ_max,opt_ (A), ${\varepsilon ¯}_{\mathrm{opt}}$ (B) and η_opt_ (C). The optimised added-shear deformation increases the mean strain (compare B with Fig. S1A) and drastically reduces η_opt_ (compare C with Fig. S1B). The red dotted curves in A–C show the maximum κ^ acquired from nine near-cyclic swimming events of a 4 dpf fish (Figs S3 and S4). The white dotted lines in A–C show the average κ^ required to make a full circle with the posterior 75% of the body.

Finally, Fig. 9 provides an overview of the results obtained for scenario 3. Fig. 9A–C shows contour plots with normalised position along the axial muscles *z* and normalised curvature κ^ as parameters. Fig. 9A shows a plot of γ_max,opt_. The largest values of γ_max,opt_ were found at 0.5*ℓ*_m_, and γ_max,opt_ increases with κ^. Fig. 9B shows a contour plot of mean muscle-fibre strain ${\varepsilon ¯}_{\mathrm{opt}}$. Strain becomes less negative towards the posterior and more negative with increasing κ^. Similar trends occur in the zero added-shear scenario (Fig. S1), but more negative mean strain values are reached with added shear across the entire parameter space because it enhances strain amplitude near the median plane. Furthermore, ${\varepsilon ¯}_{\mathrm{opt}}$ exhibits significantly less variation, as is apparent from comparing the corresponding contour plots of the coefficient of variation (Fig. 9C versus Fig. S1B). The plots show that η_opt_ and η (for scenario 2) are insensitive to κ^, but tend to increase towards the posterior.

### Strain predictions for recorded swimming motions

To assess the functional relevance of the reconstructed muscle-fibre arrangement, we derived body curvature from previously recorded near-cyclic swimming motions in larval zebrafish and focused on the most vigorous performances. The red dotted curves in Fig. 9 show the maximum curvature along the axial muscle, acquired from an ensemble of nine different near-cyclic swimming events of 4 dpf larval zebrafish, with a swimming speed of up to 60 *ℓ*_body_ s^−1^ (Fig. S4). At 0.1 of the axial muscle length *ℓ*_m_, κ^ is lowest (about 0.05), resulting in a mean strain of approximately −0.030 for the optimised added-shear case (Fig. 9B). This corresponds to a relatively small γ_max,opt_ value of about 8 deg (Fig. 9A). At 0.41*ℓ*_m_, κ^ peaks at about 0.94, corresponding to a mean strain of approximately −0.053 and γ_max,opt_=24 deg. At 0.70*ℓ*_m_, κ^ is approximately 0.085, which corresponds to a mean strain of about −0.035 and γ_max,opt_=15.4 deg.

Overall, the computed mean muscle-fibre strain falls within feasible physiological limits, although a strain of −0.053 would be quite challenging if sustained at a tail-beat frequency exceeding 90 Hz. Slightly smaller amplitudes might be used at the highest frequencies than those indicated by the red curves in Fig. 9, or the added shear might be less than the amount required to minimise strain variance. We return to this aspect in the Discussion. For the zero-added shear case (Fig. S1A), the attained mean muscle-fibre strain reaches lower amplitudes, with values of about −0.024, −0.039 and −0.028 for the discussed locations.

The horizontal white dotted lines in Fig. 9 show the κ^=0.134 level, which is required to bend the posterior 75% of the body in a full circle, assuming uniform curvature. This is presumably slightly above the maximum body curvature that larval fish can achieve. Anteriorly, this would result in a mean strain of −0.082, and posteriorly, a strain of −0.057. A smaller difference between the two locations would be achieved if body curvature slightly decreased anteriorly and increased posteriorly. Once again, the computed strain values fall within feasible physiological limits. However, at 0.5*ℓ*_m_, achieving the predicted γ_max,opt_ for a minimum strain variance of approximately 40 deg may be difficult. During such extreme deformations, minimisation of the variance in muscle-fibre strain might be compromised.

### Effect of median-plane compression on muscle-fibre strain

**Fig. 10.**
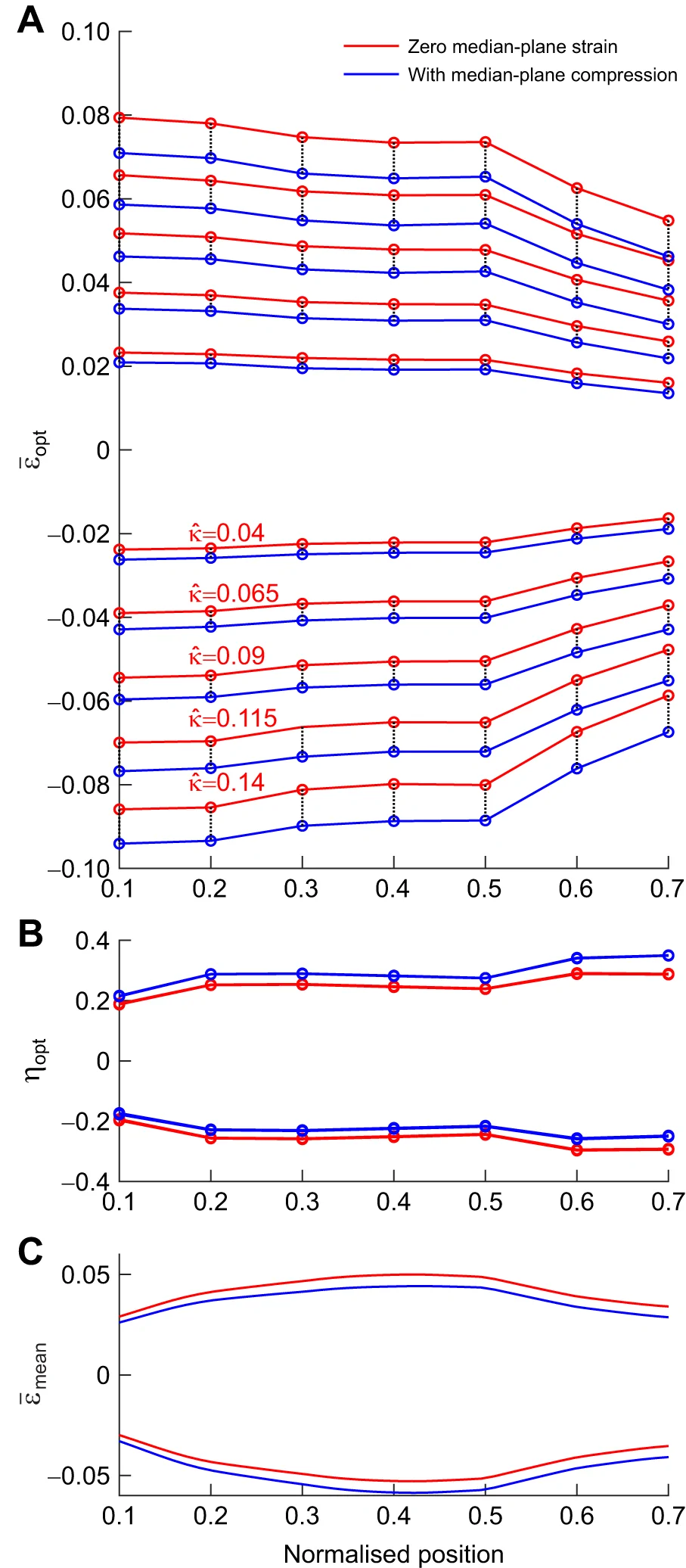
**Illustration of the effect of longitudinal compression of the median plane on mean muscle-fibre strain, coefficient of variation and maximum body curvature from kinematics of near-cyclic swimming.** Red curves represent simulations with zero strain of the median plane (scenario 3), whereas blue curves show results with a slight compression of the median plane (scenario 4). (A) Computed optimal mean strain, ${\varepsilon ¯}_{\mathrm{opt}}$, for the minimal absolute value of the coefficient of variation |η| against normalised position along the axial muscles. Bottom: family of negative strain curves for the concave body side. Black vertical lines link corresponding curves sharing the same normalised curvature κ^. Absolute strain values increase with larger κ^. Top: a similar family of positive strain curves shows results for the convex side, for the same curvature range. Median plane compression increases or decreases |${\varepsilon ¯}_{\mathrm{opt}}$| at the concave or convex body side, respectively. (B) Optimal coefficient of variation against position along the axial muscles for the convex (top) and the concave body side (bottom). The family of curves for the different body curvatures has almost collapsed to a single curve, indicating low curvature sensitivity. Median plane compression decreases or increases |η_opt_| for the concave or convex body side, respectively. (C) Mean muscle-fibre strain for the kinematics-derived maximum curvature during near-cyclic swimming for the convex (top) and the concave (bottom) body side. The bottom red curve corresponds to the red dashed curves in Fig. 9A–C.

Above, we discussed only simulations with zero strain in the median plane. However, a slight longitudinal compression of the median plane presumably occurs as a result of the generated muscle-fibre tension during swimming. Therefore, we compared the strain predictions for scenario 3 with those for scenario 4, which includes median-plane compression (Fig. 10). For the κ^-range 0.04–0.14, Fig. 10A shows that shear-optimised mean strain, ${\varepsilon ¯}_{\mathrm{opt}}$, increases in amplitude (blue versus red curves) with median plane compression along the concave side of the body, and, conversely, decreases on the convex side. Peak strain occurs at approximately 0.2*ℓ*_m_: negative on the concave side and positive on the convex side. The least negative and positive strains appear posteriorly, owing to small fibre angles near the median plane and a narrow muscle width. The absolute difference in ${\varepsilon ¯}_{\mathrm{opt}}$ between the concave and convex sides for a given combination of κ^ and location along the body is nearly the same in the two scenarios.

Fig. 10B shows families of curves of the added-shear optimised coefficient of variation η_opt_ of the sectional muscle-fibre strain. The value of |η_opt_| decreases slightly on the concave side by including median plane compression, whereas |η_opt_| increases on the convex side (Fig. 10B). For both scenarios, the smallest |η_opt_| values occur anteriorly at 0.1*ℓ*_m_, and the largest |η_opt_| is found at 0.7*ℓ*_m_. The compacted nature of the family of curves shows that η_opt_ is insensitive to changes in κ^. We conclude that the net effect of a slight median-plane compression has only a small effect on the peak-to-peak mean-strain amplitude and the relative strain variance.

Fig. 10C shows the predicted mean strain amplitude for the reconstructed curvature envelope of the nine analysed near-cyclic swimming events (Fig. S4) and its projection (red dashed curve) in the contour plot of Fig. 9B. The red curves in Fig. 10C (scenario 3) were generated by interpolating strain values along the dashed red curve, using the strain contours from Fig. 9B. These contours were themselves obtained by interpolating strain values from the 35 combinations of five curvature profiles and seven body positions. Similarly, the blue curves (scenario 4) were derived using strain values from the corresponding contour plot in Fig. S2F. As expected, scenario 4 results in larger strain amplitudes on the concave side compared with scenario 3, while the opposite is true on the convex side. In both scenarios, the largest strain amplitudes are predicted near 0.4*ℓ*_m_ along the axial muscles.

## DISCUSSION

### Functional relevance of the helical muscle-fibre arrangement during larval and juvenile development

Fast axial muscle-fibre orientation in larval zebrafish follows an arrangement of nested helices at each side of the body, one in the epaxial region and another in the hypaxial region, forming approximate mirror images (Van Raamsdonk et al., 1974; Van Leeuwen et al., 2008; Van Meer et al., 2024). If muscle fibres were instead aligned parallel to the fish's longitudinal axis, fibre strain would range from near zero in medial fibres to maximal in the lateral-most fibres, because the notochord – functioning as the central axis – is thought to be nearly incompressible. In contrast, the helical arrangement of fibre orientations is hypothesised to reduce strain variance across transverse unilateral cross-sections (Van Leeuwen et al., 2008). Approximately equal strains are likely to be advantageous, as they would enable all fibres to contribute similarly to power generation – assuming identical muscle-fibre properties and activation – and potentially allow them to operate under shared optimal strain and strain-rate conditions, regardless of their distance from the median plane. To explore this, we used a bending-beam model with added shear (Van Leeuwen et al., 2008) to examine how measured fibre orientations in the axial musculature of larval zebrafish (Van Meer et al., 2024) influence fibre-strain distributions across seven transverse body sections. The simulations covered a range of body curvatures, most within but some slightly exceeding the maximum values observed during natural swimming (Müller and Van Leeuwen, 2004; Van Leeuwen et al., 2015).

Our analysis indicates that the observed helical pattern can drastically reduce the strain variation compared with a uniform longitudinal muscle-fibre arrangement if body bending takes place in conjunction with an appropriate distribution of added shear. Added shear refers to displacement of the axial musculature in the anteroposterior direction during bending. It is most likely produced by force-producing muscle fibres near the median plane when these fibres have high azimuth and low elevation.

In 2–13 dpf zebrafish larvae, fibre azimuths near the dorsoventral level of the helix centres are close to zero, while elevations increase from the helix centre towards the median plane (Van Meer et al., 2024, 2026; see schematic diagram in Fig. S5). This arrangement reflects the geometry of nested helical trajectories with similar pitch, whose radii grow from the helix centre towards the median plane. In this central region (marked by blue lines in Fig. S5), the larval fibre pattern contributes little to added shear deformation, and added shear would do little to increase muscle-fibre strain or reduce strain variance. Along the dorsoventral axis, however, muscle fibres adjacent to the median plane show increasing azimuth and decreasing elevation with distance from the helix centre. This shift improves their ability to contract, generate power and produce added shear, which in turn helps reduce strain variance. Our 3D interpolation of muscle-fibre directions of the 4 dpf dataset illustrates such elevated medial azimuths adjacent to the dorsal and ventral sides of the horizontal septum (Fig. 3Bi–vii).

Because muscle-fibre orientation changes with dorsoventral position, the direction of induced added shear reverses when crossing the helix-centre level. Fibres with near-zero azimuth in the tissue slice between the helix centre and the median plane occupy this transition zone and are expected to experience a reduced strain range during lateral bending in swimming. This raises the question of whether the mechanical efficacy of this region is enhanced during development by adapting its geometrical pattern toward more effective muscle-fibre angles.

In zebrafish larvae between 2 and 13 dpf, the muscle-fibre arrangement in the transition zone remains relatively stable, aside from an increase in fibre number (Van Meer et al., 2026). By contrast, data from a 51 dpf juvenile (Mos and Van der Stelt, 1982) show much higher azimuths and lower elevations in this region – a configuration described as pseudo-helical by Van Leeuwen et al. (2008). At the centre of the juvenile transition zone (marked by a red line in Fig. S5), multiple parallel, closely packed connective tissue layers are present, belonging to adjacent myosepta that run approximately parallel to the horizontal septum (Van Leeuwen et al., 2008). Fibres attaching dorsally and ventrally to these connective tissue layers have opposite azimuth and elevation directions, creating a distinct jump in fibre orientation. The force-producing muscle fibres above and below these local septa probably generate added shear in opposite directions, a pattern presumably facilitated by low-friction sliding between the connective tissue layers.

The juvenile myoseptal arrangement in the transition zone is very different from that in 4 dpf larvae, where the myosepta are steeper and remain well separated. This is evident from immunohistochemical staining of anterior, mid-body and posterior myosepta in 2 and 8 dpf zebrafish, which shows a similar clear separation between neighbouring myosepta across the entire structure (see fig. 6.2 in Van Meer, 2024). The transition from the larval to the juvenile pattern therefore requires local adjustments in muscle-fibre orientation and connective tissue organisation that enable a sharp dorsoventral transition in added shear. In 4–13 dpf zebrafish larvae, this structural rearrangement is still ongoing (Van Meer et al., 2024, 2026).

The lateral nested-helix arrangement – architecturally very similar in larvae and juveniles – helps counteract the rise in muscle-fibre strain amplitude, even without added shear. This occurs because fibre elevation increases with lateral distance from the helix centre, whereas near the median plane, a high degree of added shear is required to increase strain amplitude. Consequently, the shear angle needed to minimise strain variance is highest at the median plane and declines non-linearly toward the lateral regions. Overall, the helical pattern allows for much smaller variation in muscle-fibre strain than would be possible with longitudinally oriented fibres.

The epaxial and hypaxial transition zones – more sharply defined in juveniles – constitute only a small fraction of total muscle volume, so we expected only a modest effect on the strain variance. In larvae, the notochord and neural tube occupy a relatively large proportion of the median-plane region, reducing the proportion of fibres close to this plane that would experience lower-than-average strain amplitudes. For the juvenile, Van Leeuwen et al. (2008) reported a coefficient of variation (|η_opt_|) of 0.20 at the level of the anus during high-amplitude curvature. This is approximately 33% lower than the predicted value of 0.30 for muscle section 6 of the 4 dpf larva, located near the anus.

During larval development, mechanical loading probably plays a central role in refining the helical muscle-fibre architecture required for effective locomotor performance. Consistent with this view, Van der Meulen et al. (2005) investigated the effect of muscle activity on the development of the helical muscle-fibre pattern in larval zebrafish using an immobile mutant. Although the mutant also exhibited a helical architecture, it was notably less developed than in wildtype larvae. This led the authors to propose that the initial ‘weak’ helical arrangement may be developmentally pre-specified, while mechanical feedback probably refines and reinforces fibre orientations during development. In line with this hypothesis, Van Meer et al. (2026) demonstrated that the wildtype helical muscle-fibre arrangement is already established at hatching, suggesting that constrained body movements within the chorion prior to hatching are sufficient to progress its formation beyond the initial ‘weak’ state. Post-hatching, mechanical loading during swimming probably introduces modified shear forces that drive localised adjustments in muscle-fibre orientation and connective tissue organisation. These changes may further modify muscle-tissue deformations, ultimately leading to a dynamic equilibrium between ongoing tissue development and mechanical demands. Such local self-organisation could minimise variability in muscle-fibre strain, tensile stress and power output.

Using the deformation that minimises relative strain variance, we predicted mean peak-negative muscle-fibre strain ${\varepsilon ¯}_{\mathrm{opt}}$ at the concave side during near-cyclic swimming to range from approximately −0.053 to −0.030. These strain levels correspond to maximum added shear angles of 24 deg and 8 deg, respectively, presumably falling within physiologically feasible limits. Assuming these strains apply to the fastest 4 dpf swimming event included in this study, characterised by a tail-beat frequency of 95 Hz (Van Leeuwen et al., 2015), we estimate minimum peak strain rates of −31.6 s^−1^ and −17.9 s^−1^ for sinusoidal strain profiles. Our estimates are in line with those reported by Müller and Van Leeuwen (2004) for a muscle fibre in the centre of a helical arrangement.

Delivering sufficient power for swimming at peak strain rates of up to −32 s^−1^ requires exceptionally fast muscle fibres. The experimental *in vitro* study by Mead et al. (2020) provides evidence that such fibres are present in 5 day old larval zebrafish. Their work-loop experiments – conducted by linearly stretching the pre- and post-anal regions of the body, which encompass the areas we predict to experience the highest strain and strain rates during swimming in the present study – suggest that axial muscle fibres achieve their highest mean specific power output of 134 W kg^−1^ at 80 Hz, with an 8% reduction observed at 100 Hz.

Peak power outputs recorded by Mead et al. (2020) at 80 and 100 Hz were 498.9±127 and 640.2±151.5 W kg^−1^, respectively. The latter was achieved at a measured axial strain rate of the myomeres of −18.1±2.1 s^−1^. The imposed 8% peak-to-peak sinusoidal length change in their specimens would theoretically result in a negative peak strain rate of −25.13 s^−1^. However, because of the non-uniform and dynamically varying material properties of the tissues, the resulting myomere strain waveform is skewed: the descending phase of the sine wave is longer than the ascending phase. This asymmetry explains why peak power production occurs at a less negative strain rate. Mead et al. (2020) were unable to measure the minimum strain rate with their setup. Using the measured strain rate of −18.1 s^−1^ at which peak power was observed during work-loop experiments – and assuming that peak power occurs at 40% of the minimum strain rate, Mead et al. (2020) predicted a minimum strain rate of −45 s^−1^. This value exceeds by more than threefold the minimum rate of −14.1 s^−1^ reported by Askew and Marsh (1997) for fast fibres of the mouse extensor digitorum longus muscle.

Whereas the power measurements by Mead et al. (2020) indicate the presence of superfast muscle fibres, they cannot be directly used as a reliable proxy for muscle power output during swimming, for several reasons. First, their experimental setup involved axial stretching of the preparation, whereas during swimming the body bends laterally, with only minor length changes expected along the median plane. Second, the reduced peak negative strain rate observed in their setup – compared with that of a pure sine wave – may have placed less extreme demands on the muscle fibres at high frequencies than those encountered during vigorous lateral body bending in swimming. Third, their measured myomere strain does not directly reflect *in vivo* muscle fibre strain, as the fibres are not aligned longitudinally but instead follow a nested helical arrangement. For example, if strain rates approach physiological limits for the fast, high-amplitude movements, lower strain values near the medial plane might actually enable these fibres to contribute optimally. Fourth, the muscles were artificially activated, and some fibres may have been damaged, potentially leading to suboptimal performance. Fifth, the imposed 8% length change may not have captured the global optimum for power output. Considering these experimental limitations, we expect that the maximum *in vivo* power output during swimming exceeds the values measured in the *in vitro* setup of Mead et al. (2020).

In conclusion, our analysis indicates that the close-packed helical arrangement of muscle fibres in larval zebrafish, combined with optimised added shear, reduces strain variance and supports physiologically viable strain rates during high-frequency swimming with large body curvatures. We predicted that the highest strain amplitudes and strain rates occur around 0.45*ℓ*_body_ during strenuous, near-cyclic swimming. This location along the body coincides with the predicted peak bending moments and observed maximum volume per unit length of the axial muscles (Voesenek et al., 2020). These findings suggest that – per unit length – this region produces the most mechanical work and power during swimming. Furthermore, this can be achieved at frequencies in the range of 90 to 100 Hz owing to the superfast contractile properties of the muscle fibres.

### Methodological constraints on functional interpretation

To explore how modelling assumptions influence inferred muscle-fibre strain patterns, we extended the computational framework of Van Leeuwen et al. (2008) by incorporating a slight compression of the median plane, scaled to normalised body curvature. On the concave side, this compression reduces the variation in muscle-fibre strain by allowing medial fibres with small azimuth – those that benefit relatively little from added shear – to shorten slightly more. In contrast, on the convex side, the effect is reversed because the negative influence of median-plane compression on muscle-fibre strain is largest near this plane and diminishes along the mediolateral axis. Together, the impact on a full strain cycle seems to be very small (Fig. 9), but further experimental and theoretical research is required before drawing definitive conclusions.

In the present study, we adjusted the added shear parameters to minimise strain variance across selected locations along the axial musculature and at various positions within transverse cross-sections. This approach necessitated the assumption that shear is independent of the underlying muscle-fibre orientation. However, this raises a fundamental question: how does the hypothesised shear arise from active muscle contractions in conjunction with passive mechanical structures, such as the myosepta? At present, direct measurements of shear during swimming in larval fish are unavailable and, indeed, exceedingly difficult to obtain. In the absence of a viable experimental approach, theoretical models currently offer the most reliable means of estimating muscle tissue deformation during swimming. Consequently, the extent to which larvae employ added shear to reduce strain heterogeneity remains unresolved.

The current approach relies on several simplifying assumptions. Our analysis focused exclusively on the influence of fibre orientation on muscle-fibre strain, while other contributing factors were not considered. In practice, the final optimisation may reflect a compromise between competing functional demands. By prioritising the minimisation of strain variance, we implicitly assumed that uniform contributions from all fibres within a cross-section would be optimal. However, this assumption may not hold universally. In some cases, strain heterogeneity could enhance the net bending moment or enable a broader repertoire of swimming motions.

Furthermore, by simulating strain in isolated tissue slices, we preserved volume locally but did not account for the continuum constraints imposed by the interconnected nature of the musculature. These constraints may limit the extent of permissible shear deformations and, as a result, increase the observed coefficients of variation. Strain values within a given cross-section are probably influenced by curvature dynamics in adjacent segments, depending on the wavelength of the actual body undulations. The pitch of the helical fibre arrangement may also play a critical role in redirecting forces toward the skin and the median plane, rather than solely along the longitudinal axis. In such cases, negative strain in one section could induce positive strain in a neighbouring region. Additionally, extreme azimuthal or elevational fibre angles may hinder the generation of high bending moments, further complicating the functional analysis of the muscle system.

### Functional and methodological perspectives

During swimming, added-shear deformation arises from spatially and temporally variable stress distributions in muscle fibres and connective tissue that depend on activation timing and fluid–structure interactions. Muscle fibres located halfway along one side of the body may actively induce an increasing unidirectional added-shear deformation. This process begins when the fibres are activated just before reaching their peak length on the initially convex side, and continues until they deactivate shortly before that same side reaches its maximum concave curvature, as the fibres approach their minimum length. Then, the induced shear deformation may be gradually reduced during the subsequent stretching phase when the fibres are mostly inactive. How the latter could be accomplished is less clear. Presumably, the reverse deformation depends on passive stresses in myosepta and muscle fibres, including intramuscular pressure gradients. Further posteriorly, the phase relationship between muscle activation and body curvature is expected to shift, thereby altering the mechanical stress distribution and deformation patterns. These regional differences underscore the complexity of shear generation and its modulation along the body axis during swimming. Together, these considerations highlight the limitations of current approaches for understanding shear generation during swimming.

To capture the full complexity of fish swimming, a mechanistic forward-dynamic model could be developed that incorporates muscle-fibre properties and orientations, passive tissue characteristics, muscle-activation patterns and fluid–structure interactions. Such a model would allow prediction of bending moments, swimming velocity and energetic costs as a function of muscle activation, while also accounting for the influence of muscle-fibre stress on deformation – an effect overlooked when strains are derived from prescribed kinematics. This approach would enable estimation of strain, strain rate and power output based on activation input, but requires detailed knowledge of spatially distributed activation timing, activation-dependent material properties of axial muscles and the mechanical behaviour of passive components such as the skin, myosepta and notochord. The complexity of the model and the large number of unknown parameters pose significant challenges, particularly given the difficulty of measuring the required input parameters such as muscle activation and verifying predicted tissue deformations and strain patterns in free-swimming larval fish.

### Evolutionary perspective

We hypothesise that helical axial muscle architecture in teleost larvae – probably a conserved structural adaptation – together with specialised high-performance muscle physiology have co-enabled evolutionary miniaturisation of the larval stage. These combined properties allow these animals to maintain effective undulatory swimming despite their small size, which imposes the need for high-frequency, high-amplitude body motions. By linking muscle architecture to locomotor performance and developmental constraints, this study extends current perspectives on the evolutionary biomechanics of fish larvae. More broadly, it highlights the importance of integrating biomechanical mechanisms with evolutionary theory when considering how structural and functional constraints shape organismal design. Taken together, this account suggests several avenues for future theoretical and experimental work on the interface between biomechanics, development and evolution.

## Supplementary Material

10.1242/jexbio.251951_sup1 Supplementary information
