# Supplementary material for: Predicting axial muscle-fibre strains in larval zebrafish
Source: J Exp Biol. 2026 Aug 12;229(15):jeb251951. doi: 10.1242/jeb.251951 (PMC13535098; doi:10.1242/jeb.251951)
Supplement: Supplementary file 1 [file jexbio-229-251951-s1.pdf]

Supplementary Materials and Methods

S1 Symbols, definitions, and assigned parameter values

Table S1 provides an overview of the symbols and definitions used in the main text and the Supplementary Materials. Where appropriate, assigned input values used in the simulations are provided.

Table S1: Symbols, abbreviations, and assigned values

**Symbol**	**Definition**
A_0	$A_0 = x_0\mathrm{d}z$; equation (S12)
$\mathrm{d}x_0$	distance covered by muscle-fibre element in tissue slice in transverse direction, with $\hat{\kappa} = 0$; equation (S6)
$\mathrm{d}y_0$	idem for dorsoventral distance; equation (S8))
$\mathrm{d}z$	thickness of transverse tissue slice in the reference configuration with a straight body
$\mathrm{d}z_{mp}$	thickness of transverse tissue slice at the median plane ($\mathrm{d}z_{mp} \leq \mathrm{d}z$); equation (S36)
$\mathrm{d}\phi$	infinitesimal opening angle; equation (S10)
ℓ_{body}	body length of fish
ℓ_{cn}	length of CN
$\mathrm{d}\ell_f$	length of muscle-fibre element in tissue slice; equation (S33)
$\mathrm{d}\ell_{f0}$	idem, but for $\hat{\kappa} = 0$; equation (S9)
$\ell_{m,cn}$	total axial muscle length along CN
$\ell^*_{m,cn}$	idem, but normalized; $\ell^*_{m,cn} = 1$
$\mathrm{d}\ell_{pf0}$	projected length of fibre element in frontal plane with $\hat{\kappa} = 0$; equation (S7)
ℓ_{sag}	length component of unit direction vector in sagittal plane; equation (S3)
p	exponent used to compute γ distribution; equation (4)
p_{opt}	idem, but for the η_{opt} state
q	exponent used to compute γ distribution; equation (5); $q = 0.1$ in this paper
R, $\hat{R}$	radius of lateral curvature of median plane; idem, but normalised by dividing by the maximum half-width of the axial musculature
S	transverse tissue slice
$\mathbf{v_f} = (v_x, v_y, v_z)$	unit direction vector of muscle fibre in fish frame
$\mathbf{v^*_f} = (v^*_x, v^*_y, v^*_z)$	unit direction vector of muscle fibre in CN reference frame
X, Y, Z	Cartesian frame

Journal of Experimental Biology • Supplementary information

Table S1: Symbols, abbreviations, and assigned values (continued)

Symbol	Definition
x, y, z	coordinates in fish-based reference frame
x^*, y^*, z^*	coordinates in CN reference frame; $x^* = y^* = 0$ at CN
(x_f, y_f, z_f)	centre-of-mass location of a muscle fibre
(x_f^*, y_f^*, z_f^*)	idem, but in CN reference frame; $z^* \in [0\ 1]$
x_0, y_0	the most medial location where a muscle fibre crosses the tissue-slice boundary
x_1, x_2	intersection coordinates of muscle fibre with boundaries of tissue slice
x', y', z'	coordinates in Cartesian reference frame with origin located at centre of curvature
x_1', z_1'	location of the most medial intersection with boundary of tissue slice in X', Y', Z' frame
x_2', z_2'	idem, but now for the most lateral intersection with $\gamma = 0$
x_3', z_3'	idem, but with added shear
x_{max0}	maximum width of a unilateral tissue slice in the reference configuration with $\kappa = 0$
$x_{max0,ax}$	maximum value of x_{max0} along axial muscles
y_{cn}, z_{cn}	location of a point on CN in fish-based frame
y_{ep}, y_{hyp}	the dorsoventral locations where the added shear changes sign in, respectively, the epaxial and hypaxial musculature
y_{min}, y_{max}	the most ventral and dorsal points in the transverse tissue slice
y_{low}, y_{up}	the lower and upper limits for the interval with identical sign of the added shear
$z_{m,a}, z_{m,p}$	anterior and posterior limits of the axial muscles
$z_{m,a}^*, z_{m,p}^*$	idem in CN frame
y_d, z_d	hand-digitized coordinates
α	azimuth, i.e., angle of component of unit direction vector in the frontal plane; equation (1)
β	elevation, i.e., angle of unit direction vector with the frontal plane; equation (2)
γ	added shear angle
γ_{max}; $\gamma_{max,opt}$	maximum of added shear angle; idem, but optimised for minimum coefficient of variation in muscle-fibre strain
γ_{med}	γ at median plane
δ_{cc}, δ_{cv}	angle used to compute the endpoints of muscle-fibre segments due to added shear at the concave and convex side, respectively; equations (S25) and (S29)
ε_f	longitudinal strain of a muscle-fibre element
$\bar{\varepsilon}_f$	mean value of ε_f in transverse tissue slice
ε_{mp}; $\varepsilon_{mp,min}$	longitudinal strain in the median plane; ε_{mp} at $\hat{\kappa}_{max}$; equation (S35)
$\bar{\varepsilon}_{opt}$	mean longitudinal strain of the muscle fibres in transverse tissue slice for the minimal $\lvert\eta\rvert$ case
θ	angle of muscle fibre with longitudinal direction (i.e., Z-axis)
η	coefficient of variation in muscle-fibre strain; $\eta = \sigma_\varepsilon / \bar{\varepsilon}_f$
η_{opt}	value of η that is closest to zero
ϕ	opening angle related to body curvature; $\mathrm{d}\phi = \mathrm{d}z_{mp}/R = \kappa \mathrm{d}z$
φ	sagittal projection angle; equation (S4)
ψ	angle of CN with the Z-axis
κ	lateral body curvature ($\kappa = 1/R$)
$\hat{\kappa}$; $\hat{\kappa}_{max}$	normalized lateral body curvature $\left(\hat{\kappa} = 1/\hat{R}\right)$; maximum value of $\hat{\kappa}$ used in the simulations

Journal of Experimental Biology • Supplementary information

Table S1: Symbols, abbreviations, and assigned values (continued)

Symbol	Definition
σ_ε	standard deviation in muscle-fibre strain
ξ	rotation angle of the fish frame relative to the image frame
**Abbreviations**	
CN	centre line of notochord
dpf	days post fertilization

S2 Coordinate transformation from the normalized notochord-based to the fish-based reference frame

The symbols used in this section are defined in Table S1. Previously, the locations and orientations of segmented axial muscle fibres of the 4 dpf stage were defined relative to the normalised position along and orientation of the centreline of the notochord (CN; Van Meer et al. [2024]). Van Meer et al. [2024] normalised all coordinates by dividing them by the total axial muscle length along the centreline of the notochord, $\ell_{m,nc}$. In this reference frame, let (x^*, y^*, z^*) be the coordinates in, respectively, the left-right lateral, ventrodorsal, and anteroposterior directions ($x^* = y^* = 0$ at CN; positive directions are right-lateral and dorsal; z^* denotes the position along CN and increases in the posterior direction ($z^* \geq 0$)). The position of a muscle fibre in this reference frame is expressed as (x_f^*, y_f^*, z_f^*), and its orientation with unit vector $\mathbf{v^*_f} = (v_x^*, v_y^*, v_z^*)$. The anterior and posterior borders of the axial muscles are at $z^*_{m,a} = 0$ and $z^*_{m,p} = 1$. Per definition, the normalized axial muscle length $\ell^*_{mus,nc} = z^*_{m,p} - z^*_{m,a} = 1$ in the CN frame. Previously, muscle fibres were detected only over the 95% anterior fraction of CN [Van Meer et al., 2024]. Thus, the notochord slightly extends beyond $z^*_{m,p}$.

Here, we will describe how from these data we derived the positions in the Cartesian fish-based frame, with the origin at the snout tip, the z-axis aligned longitudinally from snout tip ($z = 0$) to tail tip ($z = \ell_{body}$), and the positive X- and Y-axes pointing in the right lateral and dorsal direction, respectively. Let $z_{m,a}, z_{m,p}$ be the anterior and posterior limits of the axial muscles in the fish frame, and $\ell_m = z_{m,p} - z_{m,a}$ the axial muscle length. These correspond to $z^*_{m,a}$ and $z^*_{m,p}$ in the normalised CN frame. The range defined by $z_{m,a}, z_{m,p}$ extends anteriorly as well as posteriorly slightly beyond that of $z^*_{m,a}, z^*_{m,p}$ because of the slope of the notochord (defined by the angle $\psi = \psi(z)$ of CN with the Z-axis) at these locations.

From a lateral image of a 4 dpf fish larva, we hand-digitised the dorsal and ventral boundaries of the notochord with an in-house MATLAB function. Original pixel coordinates were transformed to coordinates in mm by using a scale bar. From the digitised (y_d, z_d) coordinates, we obtained the coordinates in the fish-based frame with the following rotation:

$$\begin{pmatrix} y \\ z \end{pmatrix} = \begin{pmatrix} y_d \cos\xi - z_d \sin\xi \\ z_d \cos\xi + y_d \sin\xi \end{pmatrix}, \tag{S1}$$

where ξ is the rotation angle of the fish frame relative to the image frame. Using the MATLAB function `spaps` (parameter settings: tol $= 2 \times 10^{-7}$, m $= 3$), we fitted smoothing quintic splines through the notochord boundaries in the fish frame. Then, we computed the coordinates of CN, (y_{cn}, z_{cn}), by computing the mean of the interpolated y-values of the upper and lower notochord boundaries at 501 equidistant locations along the z-axis, covering the entire notochord. A smoothing quintic spline function

Journal of Experimental Biology • Supplementary information

was fitted through these centre points from which the slope and angle $\psi = \psi(z_{cn})$ of the CN were derived by differentiation.

Next, we obtained $s_{cn} = s_{cn}(y_{cn}, z_{cn})$ by numerically taking the length integral along the CN. The maximum value of s_{cn} was taken as the CN length ℓ_{cn}. We normalised all coordinates in the fish-based frame by division with $\ell_{m,cn} = 0.95 * \ell_{cn}$ (the region where muscle fibres were identified), which equals the normalisation applied by Van Meer et al. [2024]. In the sequel, we will use (x, y, z) to indicate normalised coordinates in the fish-based frame, unless otherwise stated.

For the computations of the muscle-fibre strains in this paper, we need to convert the muscle-fibre locations and orientations in the CN frame to those in the fish-based frame. To do that, we fitted quintic spline functions to y_{cn} and z_{cn} as a function of z^*: the normalised distance along CN. An arbitrary position (x^*, y^*, z^*) in the axial muscles in the CN frame can now be computed as

$$\begin{pmatrix} x \\ y \\ z \end{pmatrix} = \begin{pmatrix} x^* \\ y_{cn} + y^* \cos\psi \\ z_{cn} - y^* \sin\psi \end{pmatrix}, \tag{S2}$$

where we have implemented corrections in the y- and the z-directions to account for the CN slope.

We must also correct the muscle-fibre directions if we transition from the CN frame to the fish-based frame. Hence, from the unit direction vectors (v_x^*, v_y^*, v_z^*) in the CN frame, we derived the equivalent unit vectors (v_x, v_y, v_z) in the fish-based frame. First, we define the magnitude of the unit-vector projection onto the sagittal plane, denoted ℓ_{sag}, as

$$\ell_{sag} = \sqrt{v_y^{*2} + v_z^{*2}}, \tag{S3}$$

and the sagittal projection angle φ as

$$\varphi = \arcsin\left(\frac{v_y^*}{\ell_{sag}}\right). \tag{S4}$$

Then, we can compute the unit direction vector in the fish-based frame as

$$\begin{pmatrix} v_x \\ v_y \\ v_z \end{pmatrix} = \begin{pmatrix} v_x^* \\ \ell_{sag}\sin(\varphi + \psi) \\ \ell_{sag}\cos(\varphi + \psi) \end{pmatrix}. \tag{S5}$$

Finally, we made a linear orthogonal fit through the 501 CN locations using the MATLAB function linortfit2 by Carr [2024]. The resulting slope differed by less than 0.1° from the orientation of the Z-axis.

S3 Computation of muscle-fibre strains

Here, we provide the mathematical background for the strain computations. Symbols are listed in Table S1. The initial infinitesimal thickness of the tissue slice is $\mathrm{d}z$. At the neutral plane, $\mathrm{d}z$ is assumed to be constant during lateral bending. The neutral plane overlaps with the median plane if the latter is incompressible. The neutral plane shifts to the convex side during lateral body bending if the median plane is longitudinally compressed (i.e., is shortened longitudinally). A muscle fibre that traverses a transverse tissue slice S of infinitesimal thickness $\mathrm{d}z$ in the reference configuration covers a distance in the slice in the transverse direction $\mathrm{d}x_0$ (Fig. 2**B1**):

$$\mathrm{d}x_0 = \mathrm{d}z \tan\alpha. \tag{S6}$$

Journal of Experimental Biology • Supplementary information

In the reference position, the coordinates in the transverse direction of the start and endpoints of the muscle-fibre segment on the anterior and posterior slice boundaries are given by $(x_0, x_0 + \mathrm{d}x_0)$. The projected fibre length onto the frontal plane is

$$\mathrm{d}\ell_{pf0} = \mathrm{d}z/\cos\alpha, \qquad -\pi/2 < \alpha < \pi/2. \tag{S7}$$

The covered dorsoventral distance is:

$$\mathrm{d}y_0 = \mathrm{d}\ell_{pf0}\tan\beta, \qquad -\pi/2 < \beta < \pi/2, \tag{S8}$$

and the length of the fibre element is:

$$\mathrm{d}\ell_{f0} = \mathrm{d}\ell_{pf0}/\cos\beta, \qquad -\pi/2 < \beta < \pi/2. \tag{S9}$$

If the fish median plane bends locally into a circular arc with radius R, then the infinitesimal opening angle is:

$$\mathrm{d}\phi = \mathrm{d}z_{mp}/R, \tag{S10}$$

where $\mathrm{d}z_{mp}$ is the thickness of the tissue slice at the median plane. For lateral bending, we assume $\mathrm{d}z_{mp} = \mathrm{d}z$ for an incompressible median plane, whereas $\mathrm{d}z_{mp} \leq \mathrm{d}z$ for a compressible median plane. The radius R varies along the longitudinal axis of the fish. We assume, however, that R varies so slowly with z that for the present purposes we can take a constant value of R for the considered infinitesimally thin tissue slice. The curvature of the median plane is defined as $\kappa = 1/R$, where $\kappa(z,t)$ is a function of time and longitudinal location. We define the normalized curvature as:

$$\hat{\kappa} = \kappa x_{max0,ax}, \tag{S11}$$

where $x_{max0,ax}$ is the maximum half width of the axial musculature (here approximated as the maximum value of x of the interpolated muscle-fibre locations in the considered seven sections along the axial musculature).

We assume that lateral thickening occurs on the concave side of the body, with no alteration in the dorsoventral position of material points (type I deformation, as previously defined by Van Leeuwen et al. [2008]). Vice versa, with zero strain in the median plane, lateral thinning occurs at the convex side. However, if the median plane is longitudinally compressed, a slight lateral thickening occurs at the convex side in a thin layer between the neutral and median planes; lateral to said layer, thinning occurs. We do not consider a deformation with constant distances of the material points to the median plane (type II deformation, see Van Leeuwen et al. [2008]) because it leads to similar results for the strain calculations.

We cannot use the *in vivo* geometry changes to verify our deformation assumptions because measuring internal deformations in larval fish is extremely challenging. However, if our conclusions about the functional significance hold for both extremes, they are likely to be relevant to the *in vivo* situation as well.

To calculate the longitudinal muscle-fibre strain, we need to determine the new locations of the endpoints of the infinitesimal muscle-fibre segment because of the lateral body curvature. Let the fibre segment extend between x_0 and $x_0 + \mathrm{d}x_0$ in the (lateral) X-direction, and infinitesimal area element A_0 in the (x, z) plane (see Fig. 2**B2**) be defined as:

$$A_0 = x_0\mathrm{d}z. \tag{S12}$$

Journal of Experimental Biology • Supplementary information

Assuming incompressible muscle tissue, A_0 should be constant. In the deformed state at the concave body side, A_0 can be expressed as:

$$A_0 = \left(R^2 - (R - x_1)^2\right) \mathrm{d}\phi/2 = \left(R^2 - (R - x_1)^2\right) \mathrm{d}z_{mp}/\left(2R\right), \tag{S13}$$

where x_1 is the distance between the medialmost (first) endpoint of the fibre element and the median plane at the concave side of the body ($x_1 > 0$). We define x_2 as the distance between the lateralmost endpoint of the fibre element and the median plane. From equations (S12) and (S13), the locations where the fibre crosses the slab can be computed:

$$x_1 = R - \sqrt{R^2 - \frac{2Rx_0\mathrm{d}z}{\mathrm{d}z_{mp}}}, \tag{S14}$$

and

$$x_2 = R - \sqrt{R^2 - \frac{2R\left(x_0 + \mathrm{d}x_0\right)\mathrm{d}z}{\mathrm{d}z_{mp}}}. \tag{S15}$$

With an incompressible median plane (i.e., $\mathrm{d}z_{mp} = \mathrm{d}z$), these equations are, respectively, reduced to

$$x_1 = R - \sqrt{R^2 - 2Rx_0}, \qquad x_0 \leq R/2 \tag{S16}$$

and

$$x_2 = R - \sqrt{R^2 - 2R\left(x_0 + \mathrm{d}x_0\right)}, \qquad x_0 + \mathrm{d}x_0 \leq R/2. \tag{S17}$$

For the convex side, we can derive a similar set of equations. In the deformed state, A_0 can now be expressed as:

$$A_0 = \left((R + x_1)^2 - R^2\right) \mathrm{d}\phi/2 = \left((R + x_1)^2 - R^2\right) \mathrm{d}z_{mp}/\left(2R\right), \tag{S18}$$

where x_1 is the distance between the medialmost (first) endpoint of the fibre element and the median plane ($x_1 > 0$). We define x_2 as the distance between the lateralmost endpoint of the fibre element and the median plane. From equations (S12) and (S18), the locations where the fibre crosses the slab can be computed:

$$x_1 = \sqrt{\frac{2Rx_0\mathrm{d}z}{\mathrm{d}z_{mp}} + R^2} - R, \tag{S19}$$

and

$$x_2 = \sqrt{\frac{2R\left(x_0 + \mathrm{d}x_0\right)\mathrm{d}z}{\mathrm{d}z_{mp}} + R^2} - R, \tag{S20}$$

With an incompressible median plane (i.e., $\mathrm{d}z_{mp} = \mathrm{d}z$), these equations are, respectively, reduced to

$$x_1 = \sqrt{2Rx_0 + R^2} - R \tag{S21}$$

and

$$x_2 = \sqrt{2R\left(x_0 + \mathrm{d}x_0\right) + R^2} - R. \tag{S22}$$

Journal of Experimental Biology • Supplementary information

For the calculation of muscle-fibre strain, we establish a local Cartesian frame of reference (x', y', z'), with its origin at the centre of curvature, and y' aligned with y. We position $y' = y = 0$ midway between the dorsalmost point of the tissue slab ($y' = y = y_{max}$) and the ventralmost point ($y' = y = y_{min}$). The positive x'-axis is assumed to pass through the centre of the medial boundary of the considered muscle segment, while the z'-axis runs parallel to the tangent of the centre of this medial boundary. Figure 4F of Van Leeuwen et al. [2008] shows a projection in a horizontal plane, with a constant y'. For the concave side, the (x', z')-position of the medial endpoint (point 1) is given by:

$$(x'_1, z'_1)_{cc} = \left((R - x_1) \cos \frac{\mathrm{d}\phi}{2}, (R - x_1) \sin \frac{\mathrm{d}\phi}{2} \right). \tag{S23}$$

The position of the second endpoint is more complicated to derive, as we allow for an added shear deformation in planes with a constant value of y'. Let (x'_2, z'_2) be the position of the second endpoint without added shear (i.e., simple beam deformation):

$$(x'_2, z'_2)_{cc} = \left((R - x_2) \cos \frac{\mathrm{d}\phi}{2}, -(R - x_2) \sin \frac{\mathrm{d}\phi}{2} \right). \tag{S24}$$

In the derivation of $(x'_1, z'_1)_{cc}$ and $(x'_2, z'_2)_{cc}$, we assumed symmetry with the infinitesimal fibre element symmetrically distributed about the mid-angle, with its endpoints located at angular positions $\pm\mathrm{d}\phi/2$. Due to the shear deformation with local shear angle γ, the second endpoint of the muscle-fibre segment needs to be corrected. This can be done by correcting the half-opening angle $\mathrm{d}\phi/2$ with angle δ_{cc} due to shear angle γ. Let (x'_3, z'_3) represent the shifted location resulting from the local added shear. To derive the strain in the muscle-fibre segment, we must calculate (x'_3, z'_3). For the concave body side, the shifted position should lie on the circle with radius $(R - x_2)$, centred around the y'-axis. Using the law of sines, angle δ_{cc} can be computed from the known values of R, x_1, x_2, and γ:

$$\delta_{cc} = -\gamma + \arcsin \left(\frac{R - x_1}{R - x_2} \sin \gamma \right). \tag{S25}$$

Using this solution for δ_{cc}, we can now compute the location of the shifted endpoint:

$$(x'_3, z'_3)_{cc} = \left((R - x_2) \cos \left(\frac{\mathrm{d}\phi}{2} - \delta_{cc} \right), (R - x_2) \sin \left(\frac{\mathrm{d}\phi}{2} - \delta_{cc} \right) \right). \tag{S26}$$

With $\gamma > 0$ and $\delta_{cc} > 0$, the distance between $(x'_1, z'_1)_{cc}$ and $(x'_3, z'_3)_{cc}$ will be smaller than the distance between $(x'_1, z'_1)_{cc}$ and $(x'_2, z'_2)_{cc}$, yielding a negative strain of the muscle-fibre segment. For muscle fibres aligned with the longitudinal body axis (main text: scenario 1), $x_2 = x_1$, which implies $\delta_{cc} = 0$, and $(x'_3, z'_3)_{cc} = (x'_2, z'_2)_{cc} = (x'_1, z'_1)_{cc}$, irrespective of the value of γ. In other words, for longitudinally aligned fibres ($x_1 = x_2$), both endpoints remain at the same radial distance from the centre of curvature, such that the shear-induced angular correction vanishes and the endpoint positions are unchanged. Consequently, shear does not contribute to muscle-fibre strain in this configuration.

Similarly, for the convex side, we derive, respectively:

$$(x'_1, z'_1)_{cv} = \left((R + x_1) \cos \frac{\mathrm{d}\phi}{2}, (R + x_1) \sin \frac{\mathrm{d}\phi}{2} \right) \tag{S27}$$

and

$$(x'_2, z'_2)_{cv} = \left((R + x_2) \cos \frac{\mathrm{d}\phi}{2}, -(R + x_2) \sin \frac{\mathrm{d}\phi}{2} \right). \tag{S28}$$

Let $(x'_3, z'_3)_{cv}$ represent the shifted location resulting from the local added shear angle γ, at the convex

Journal of Experimental Biology • Supplementary information

body side. To derive the strain in the muscle-fibre segment, we must calculate (x_3', z_3'). The shifted position should lie on the circle with radius $(R + x_2)$, centred around the y'-axis. Using the law of sines and given values of shear angle γ (considered positive for the anticlockwise direction), R, x_1, and x_2, it follows that

$$\delta_{cv} = \gamma - \arcsin\left(\frac{R + x_1}{R + x_2}\sin\gamma\right), \tag{S29}$$

where δ_{cv} is the correction angle for $\mathrm{d}\phi/2$ due to shear. Using this result, we can compute the shifted location of the second endpoint of the muscle-fibre segment:

$$(x_3', z_3')_{cv} = \left((R + x_2)\cos\left(\frac{\mathrm{d}\phi}{2} + \delta_{cv}\right), (R + x_2)\sin\left(\frac{\mathrm{d}\phi}{2} + \delta_{cv}\right)\right). \tag{S30}$$

With $\gamma_{cv} > 0$ and $\delta_{cv} > 0$, the distance between $(x_1', z_1')_{cv}$ and $(x_3', z_3')_{cv}$ will be larger than the distance between $(x_1', z_1')_{cv}$ and $(x_2', z_2')_{cv}$, yielding a positive effect on the strain of the muscle-fibre segment. Analogous to the concave side, $x_1 = x_2$ implies $\delta_{cv} = 0$, such that added shear does not contribute to the strain in longitudinally oriented muscle fibres. The x'-and z'-differences of the positions of the endpoints of the fibre element are:

$$\mathrm{d}x' = x_3' - x_1', \tag{S31}$$

$$\mathrm{d}z' = z_3' - z_1'. \tag{S32}$$

The length of the fibre element can now be calculated as:

$$\mathrm{d}\ell_f = \sqrt{\mathrm{d}x'^2 + \mathrm{d}y_0^2 + \mathrm{d}z'^2}. \tag{S33}$$

Finally, we obtain the longitudinal strain of the fibre element:

$$\varepsilon_f = (\mathrm{d}\ell_f - \mathrm{d}\ell_{f0})/\mathrm{d}\ell_{f0}. \tag{S34}$$

Given a specified curvature of the median plane, along with a prescribed shear angle distribution $\gamma(x, y)$ and measured fibre-angle distributions $\alpha(x, y)$ and $\beta(x, y)$, the muscle-fibre strains can now be calculated as a function of (x, y, R) or $(x, y, \hat{\kappa})$ across the transverse muscle slab. This enables us to assess the impact of muscle architecture on the strain distribution. In our simulations, we used $\hat{\kappa}$ values of, respectively, 0.04, 0.065 0.09, 11.5, and 0.14. For the simulations of a compressible median plane, we assumed:

$$\varepsilon_{mp} = \frac{\hat{\kappa}\varepsilon_{mp,min}}{\hat{\kappa}_{max}}, \tag{S35}$$

where $\hat{\kappa}_{max} = 0.14$ is the maximum normalised curvature used in the simulations and $\varepsilon_{mp,min}$, the corresponding, prescribed, longitudinal strain in the median plane. Equation (S35) can subsequently be used to compute $\mathrm{d}z_{mp}$:

$$\mathrm{d}z_{mp} = \mathrm{d}z\,(1 + \varepsilon_{mp}). \tag{S36}$$

The central and largest region in the axial muscles, delimited by $[y_{hyp}, y_{ep}]$, is located directly dorsal and ventral to the horizontal septum. It encompasses roughly the ventral half of the epaxial muscle-fibre trajectories and the dorsal half of the hypaxial muscle-fibre trajectories. In this area, muscle fibres run from the median plane in a posterolateral direction. The shortening of these fibres at the concave body side

is amplified by a positive value of γ, according to our sign convention. The counterclockwise orientation of the epaxial trajectories and the clockwise orientation of the hypaxial trajectories produce a positive γ when contracting on the concave side of the body, causing an anterior shift in the lateral portions of the muscle slab (similar to the tissue deformation shown in Fig. 1B1). This anterior movement in a series of connected myomeres supports the lateral motion of the caudal peduncle and tail fin toward the concave side of the body.

In the region delimited by $[y_{ep1}, y_{max}]$, the muscle fibres run in the posteromedial direction. Here, a negative value of γ increases muscle-fibre shortening close to the median plane at the concave side. Consequently, muscle material in this area is expected to shift posteriorly relative to the median plane. A similar effect occurs in the region where $y \in [y_{min}, y_{hyp}]$. Fig. 2C shows an example of a γ-distribution.

S4 Supplementary figures

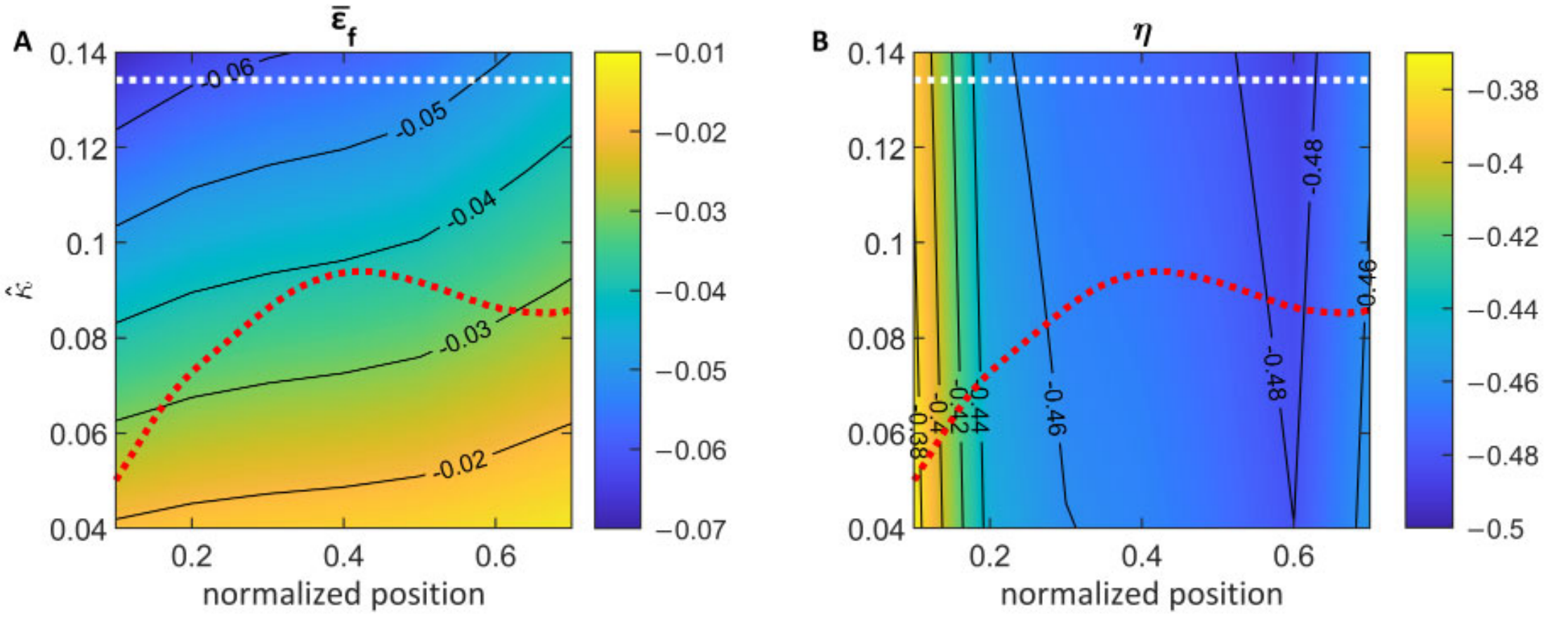

Fig. S1: Overview of mean muscle-fibre-aligned strains and coefficient of variation for scenario 2 (using reconstructed muscle-fibre orientations but without added shear deformation, i.e., $\gamma_{max} = 0$). (A) Contour plot of $\bar{\varepsilon}_f$, showing that $\bar{\varepsilon}_f$ increases with a higher curvature, and decreases in the posterior direction for a given value of $\hat{\kappa}$. (B) Contour plot of η. Overall, η varies very little with changes in $\hat{\kappa}$. The red dotted curves in (A)–(B) show the maximum $\hat{\kappa}$ acquired from nine near-cyclic swimming events of a 4 dpf fish (Figs. S3, S4). The white dotted lines in (A)–(B) show the average $\hat{\kappa}$ required to make a full circle with the posterior 75% of the body.

Journal of Experimental Biology • Supplementary information

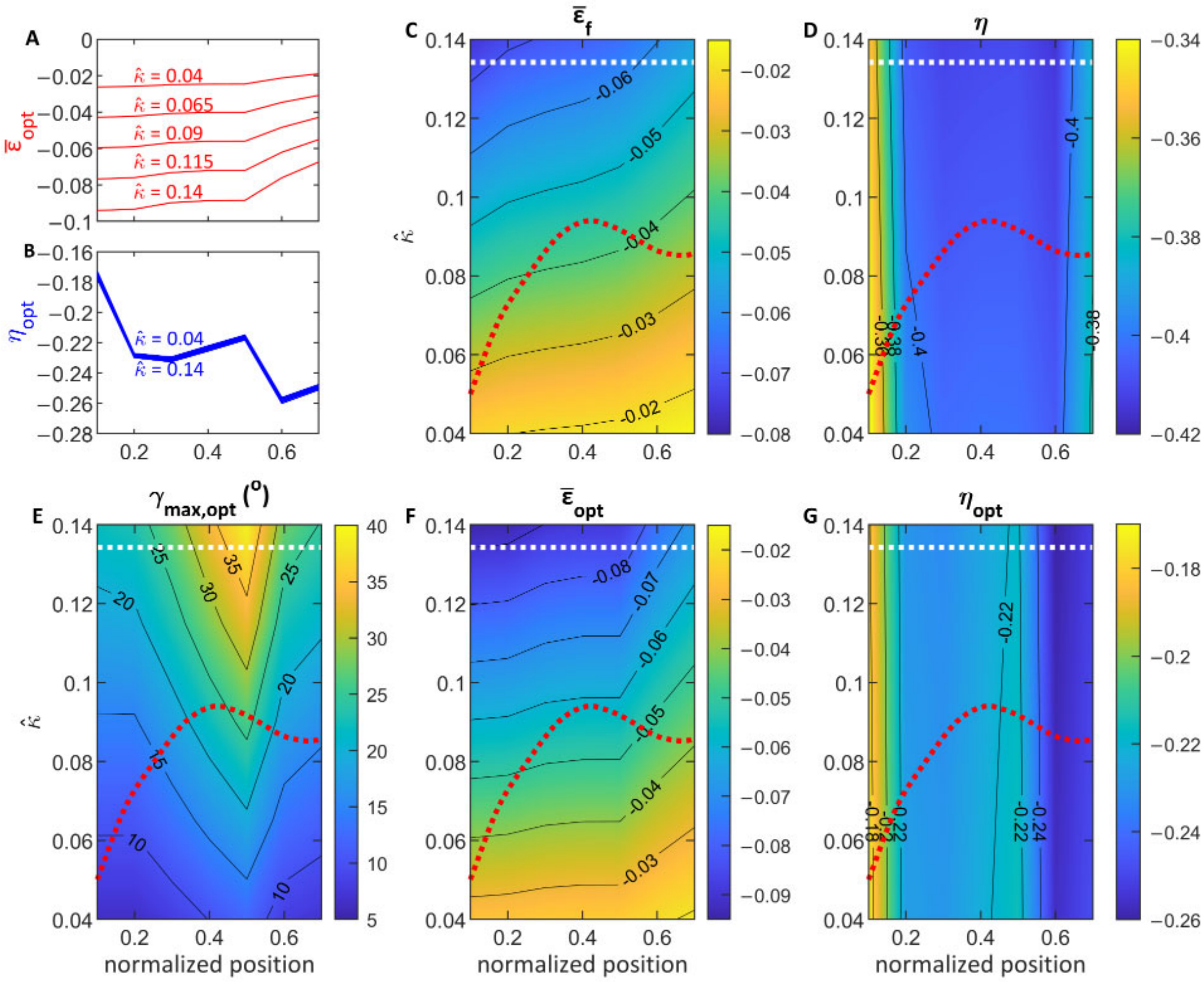

Fig. S2: Overview of the effect of normalised curvature ($\hat{\kappa}$) and location along axial muscles on the optimised maximum added shear ($\gamma_{max,opt}$), mean muscle-fibre strain ($\bar{\varepsilon}_{opt}$), and coefficient of variation (η_{opt}) for minimal relative strain variation (scenario 4: panels (A,B) and (E–G)). In contrast to the deformation in scenario 3 (Fig. 9), a compression of the median plane was implemented, with a minimum longitudinal strain of 0.01 at the maximum normalised curvature of 0.2. Anteroposterior location is normalised for axial muscle length. (A,B) Families of curves for $\bar{\varepsilon}_{opt}$ (red) and η_{opt} (blue) for different values of $\hat{\kappa}$. (C) Contour plot of $\bar{\varepsilon}_f$ without added shear (i.e., $\gamma_{max} = 0$), showing that $\bar{\varepsilon}_f$ increases with a higher curvature, and decreases in the posterior direction for a given value of $\hat{\kappa}$. (D) Contour plot of η for the zero added-shear case. Results in panels (C) and (D) resemble scenario 2, but with median plane compression. Overall, η varies very little with changes in $\hat{\kappa}$. (E) Contour plot of $\gamma_{max,opt}$ for the optimised added-shear condition. Panels (F) and (G) are similar to panels (C) and (D), but now for the optimised added-shear condition, showing contours of $\bar{\varepsilon}_{opt}$ and η_{opt}. The optimised added-shear deformation increases the mean strains (compare panels (C) and (F) and drastically reduces η_{opt} (panels (D) and (G)). The red dotted curve (panels (C)–(G)) shows the maximum $\hat{\kappa}$ acquired from nine near-cyclic swimming events of a 4 dpf fish (Figs. S3, S4). The white dotted line (panels (C)–(G)) shows the average $\hat{\kappa}$ required to make a full circle with the posterior 75% of the body.

Journal of Experimental Biology • Supplementary information

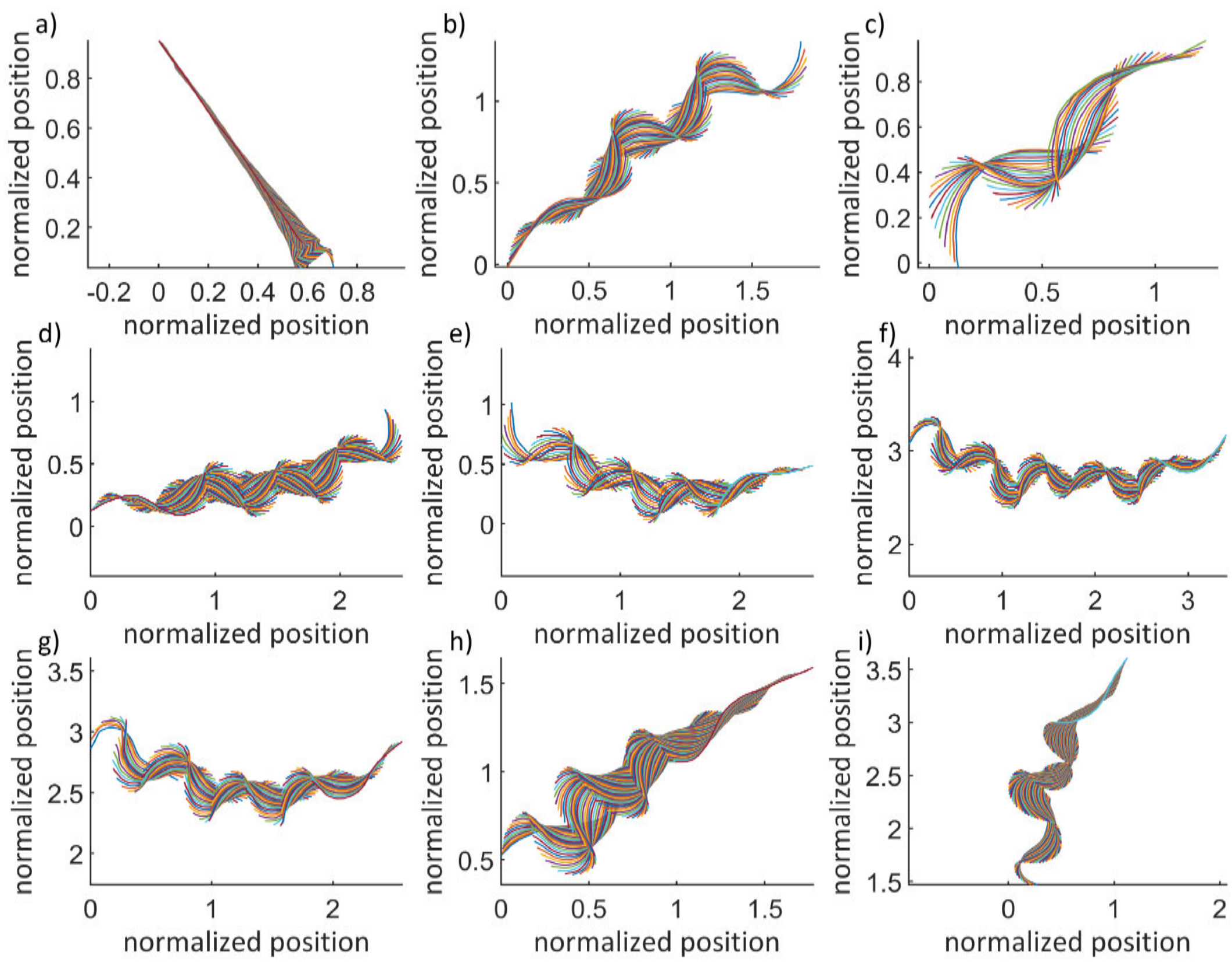

Fig. S3. Digitized midlines of nine near-cyclic swimming events of 4 dpf larval zebrafish. Time interval between successive midlines is 0.5 ms. Positions are normalized relative to body length, ℓ_{body}. These swimming events were used to estimate maximum curvatures along the axial muscles (see dotted white curves in Figs. 9A-C, S2C-G). Based on Van Leeuwen et al. [2015]

Journal of Experimental Biology • Supplementary information

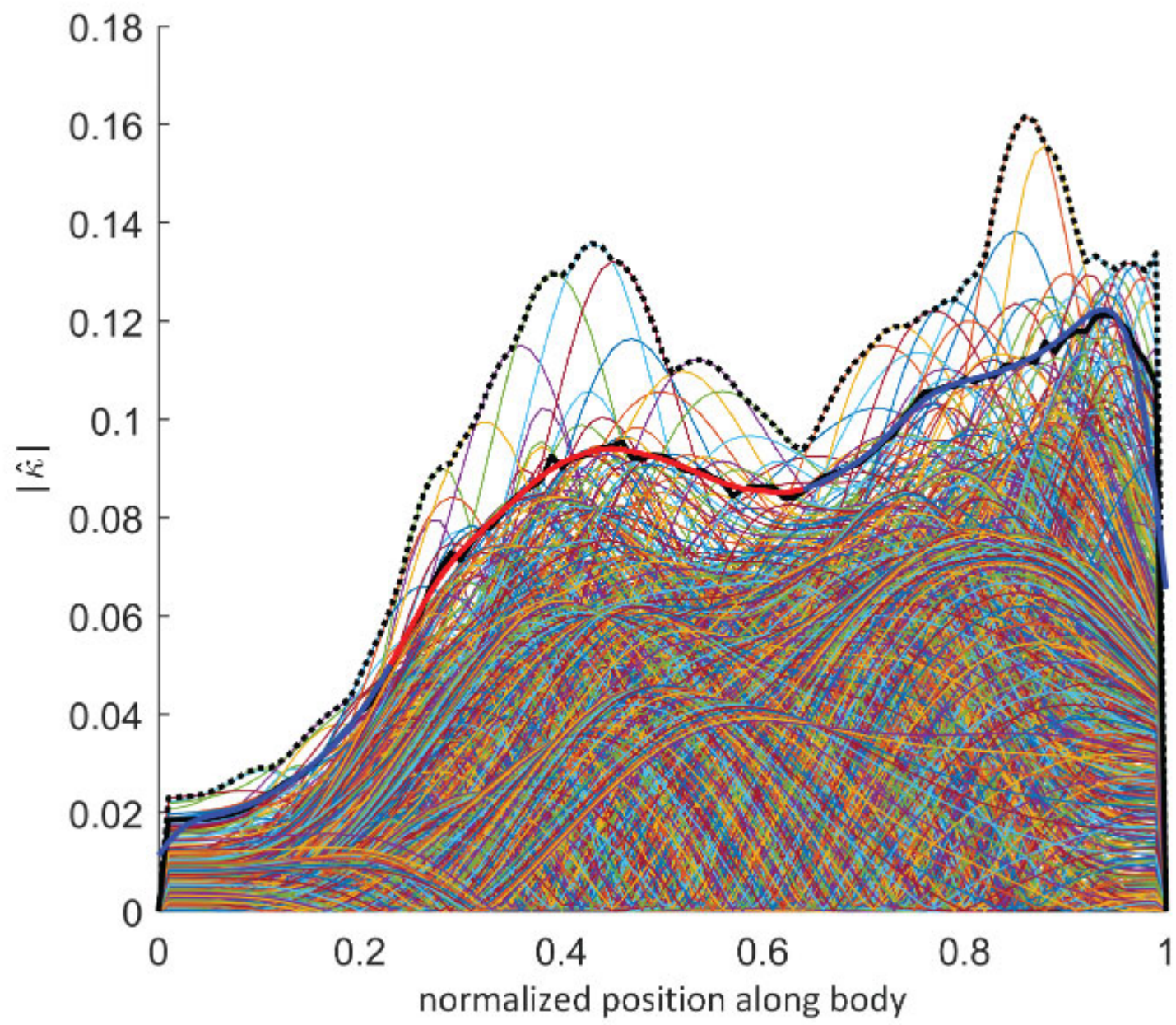

Fig. S4: Computed normalized absolute curvatures ($|\hat{\kappa}|$)of the nine swimming events shown in Fig. S3 (thin lines). The dotted black curve shows the curvature envelope of all swimming events. The heavy black curve shows the 99% curvature envelope with the 1% highest values eliminated for each location. The heavy blue curve shows a smoothing cubic spline fit of the 99% envelope. The red heavy curve shows the smoothed 99% envelope overlapping with the seven slices of axial muscle along the body that were used to compute muscle-fibre strains.

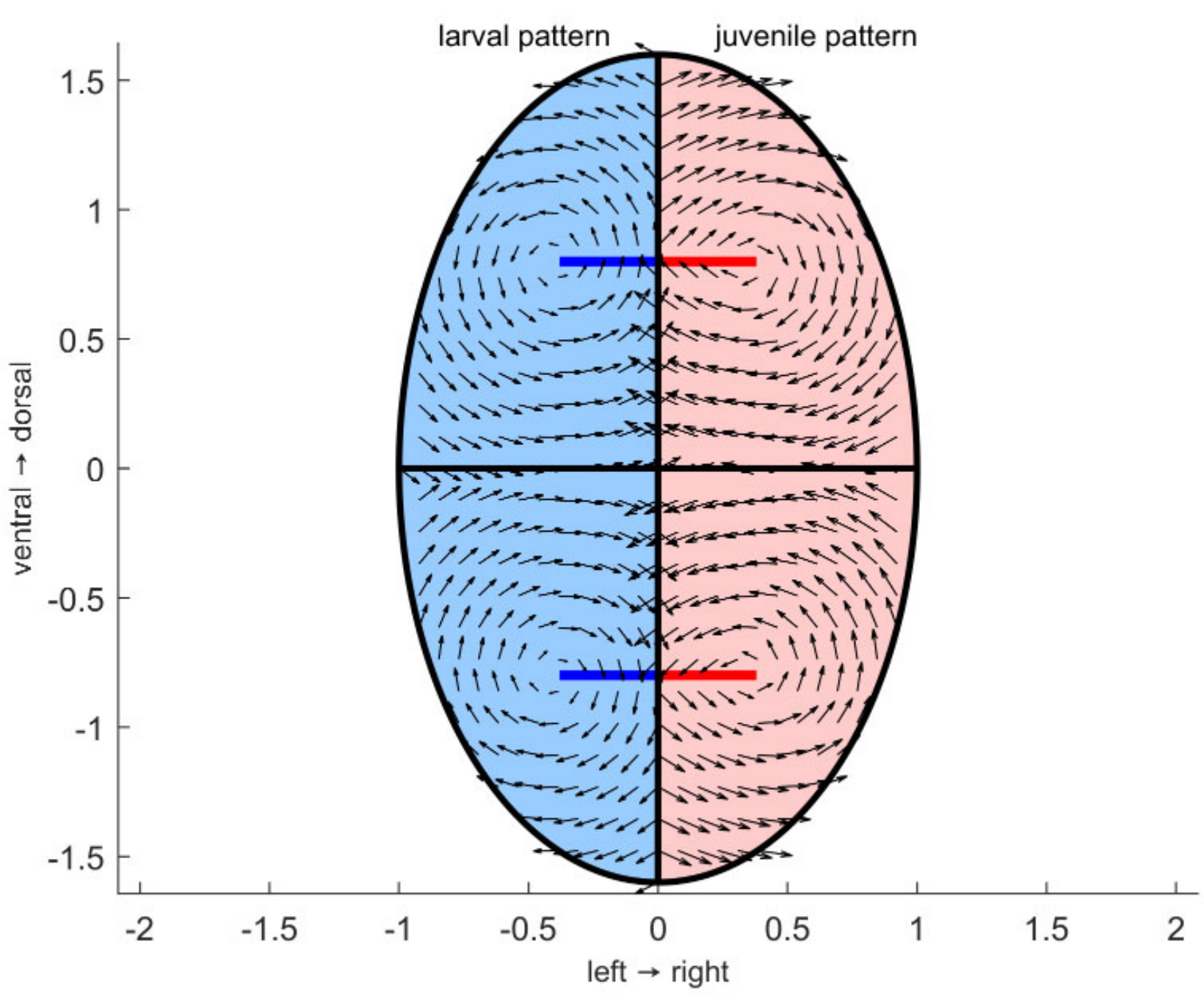

Fig. S5: Simplified schematic illustrating typical muscle-fibre arrangements in larval (blue) and juvenile (pink) stages, with direction vectors projected onto the transverse plane. Horizontal blue and red lines mark the locations of transition zones discussed in the main text.

Journal of Experimental Biology • Supplementary information

References

F. Carr. Orthogonal Linear Regression (https://www.mathworks.com/matlabcentralfileexchange/16800-orthogonal-linear-regression). *MATLAB Central File Exchange*, Retrieved: 15 July 2024, 2024.

J. L. Van Leeuwen, T. Van der Meulen, H. Schipper, and S. Kranenbarg. A functional analysis of myotomal muscle-fibre reorientation in developing zebrafish *Danio rerio*. *Journal of Experimental Biology*, 211(8): 1289–1304, 2008. doi: 10.1242/jeb.012336.

J. L. Van Leeuwen, C. J. Voesenek, and U. K. Müller. Data from: How body torque and Strouhal number change with swimming speed and developmental stage in larval zebrafish, 2015, Dryad Digital Repository: http://doi.org/10.5061/dryad.r503m.

N. M. M. E. Van Meer, J. L. Van Leeuwen, H. Schipper, and M. J. Lankheet. Axial muscle-fibre orientations in four days post-fertilization larval zebrafish. *Journal of Anatomy*, 246:517–533, 2024. doi: 10.1111/joa.14161.
